# Distribution of nicotinamide phosphoribosyltransferase in genus *Aggregatibacter* and description of *Aggregatibacter animalimorsus* sp. nov., *Aggregatibacter holmi* sp. nov., and *Aggregatibacter valvarum* sp. nov.

**DOI:** 10.3389/fmicb.2026.1866808

**Published:** 2026-07-29

**Authors:** Anne A. Thomsen, Anne B. Jensen, Dennis S. Hansen, Martin V. Møller, Niels Nørskov-Lauritsen

**Affiliations:** 1 Department of Clinical Microbiology, Aarhus University Hospital, Aarhus, Denmark; 2 Department of Dentistry and Oral Health, Aarhus University, Aarhus, Denmark; 3 Department of Clinical Microbiology, Copenhagen University Hospital, Herlev, Denmark; 4 Department of Clinical Microbiology, Esbjerg Hospital, University Hospital of Southern Denmark, Esbjerg, Denmark

**Keywords:** clinical significance, commensals, core genes, NAD, species definition

## Abstract

**Introduction:**

Bacteria of genus *Aggregatibacter* vary with respect to dependence on exogenous nicotinamide adenine dinucleotide (NAD). The genus is incompletely resolved, as 20% of deposited *Aggregatibacter* genome sequences do not belong to named species.

**Methods:**

We performed phenotypic analysis of the four named *Aggregatibacter* species plus four additional genomospecies, and bioinformatics analysis of 282 genomes; 237 of these genomes represent cultured isolates.

**Results:**

Independence of NAD is ubiquitous in genomospecies related to *Aggregatibacter actinomycetemcomitans*, common in *Aggregatibacter aphrophilus* and *Aggregatibacter kilianii*, and absent from *Aggregatibacter segnis* plus the six additional genomospecies. Characterised genomospecies qualify for nomenclatural approval according to phenotype and accepted genomic boundaries. Suggested species *Aggregatibacter valvarum* has clinical relevance, as three of five isolates were cultured from human blood.

**Conclusion:**

Naming of three species is proposed. A total of seven named *Aggregatibacter* species will comprise 87% of deposited genome sequences from cultured strains. The benefit of naming of three other genomospecies is uncertain. Two genomospecies cannot be validly named until *in vitro* culture has been accomplished.

## 1 Introduction

Many bacteria like, e.g., *Enterobacteriaceae* make use of a wide range of gene products to control biosynthesis and recycling of nicotinamide adenine dinucleotide (NAD), whereas all *Pasteurellaceae* acquire this essential nutrient from their environment, either as NAD or as a limited number of precursors (Gerlach and Reidl, 2006; Nørskov-Lauritsen, 2014). Ample nicotinamide is present in complex media, and strains of *Pasteurellaceae* with a functional nicotinamide phosphoribosyltransferase do not show satellitism around producer colonies of other bacterial species (“V factor-” or NAD-dependence) when cultured on blood agar plates. When genus *Aggregatibacter* was created in 2006, it combined a species independent of NAD (*Aggregatibacter actinomycetemcomitans*; previously affiliated with *Actinobacillus*), a species that exhibits satellitism (NAD-dependence) (*Aggregatibacter segnis*; previously affiliated with *Haemophilus*), and a species with both NAD-dependent and NAD-independent isolates (*Aggregatibacter aphrophilus*; previously designated *Haemophilus aphrophilus* for NAD-independent isolates and *Haemophilus paraphrophilus* for NAD-dependent isolates) (Nørskov-Lauritsen and Kilian, 2006). In 2018, *Aggregatibacter kilianii* was added; one of 10 characterised isolates of *A. kilianii* was NAD-dependent (Murra et al., 2018).

Another important characteristic of *Aggregatibacter* is the conspicuous reliance of carbon dioxide for growth on infusion agars. When Omar Khairat described *Haemophilus aphrophilus* from a fatal case of infective endocarditis in 1940, the striking dependence on CO_2_ was considered for naming. In the absence of a root word signifying CO_2_, Khairat focused on a manifestation of CO_2_ well known in classical time, the formation of bubbles of gas in fermenting wine, the froth (*aphros*) (Khairat, 1940). Per Holm later documented that *Actinobacillus actinomycetemcomitans* required more CO_2_ than present in atmospheric air to grow on the surface of solid media, irrespective of aerobic or anaerobic conditions (Holm, 1954). Because culture of clinical samples on enriched media in 5% CO_2_ has been standard procedure for many years, CO_2_-dependence is rarely characterised when unusual, fastidious bacteria are reported.

Clinically, genus *Aggregatibacter* encompasses human commensals occasionally associated with specific inflammations and infections, such as aggressive periodontitis, endocarditis and brain abscess (Haubek et al., 2008; Kommedal et al., 2014; Nørskov-Lauritsen et al., 2019; Andersen et al., 2022). The genus is part of the HACEK bacteria (*Haemophilus*, *Aggregatibacter*, *Cardiobacterium*, *Eikenella* and *Kingella*) (Geraci and Wilson, 1982), responsible for 1–3% of cases of infective endocarditis. *A. actinomycetemcomitans* and *Haemophilus parainfluenzae* are the two most common HACEK bacteria causing this infection (Yew et al., 2014; Lützen et al., 2018). Sporadically, *A. actinomycetemcomitans* are cultured from primates (Karched et al., 2012).

Speciation of taxa in genus *Aggregatibacter* is incomplete. Multilocus Enzyme Electrophoresis (MLEE) of *A. actinomycetemcomitans* identified a group of serotype e isolates (later referred to as clade e’) that were clearly separate from the core of the species (Poulsen et al., 1994); these isolates carry the unusual 16S rRNA type V sequence (Kaplan et al., 2002). Reijden and coworkers characterised 18 strains of this clade from Java, Netherland and Switzerland, and demonstrated the specific inability to ferment starch and glycogen (van der Reijden et al., 2010). Finally, the clade e’ outgroup has been documented by genome sequencing (Jorth and Whiteley, 2012; Kittichotirat et al., 2016; Nedergaard et al., 2019). Other branches of genus *Aggregatibacter* are less well studied. Aberrant isolates of *Haemophilus paraphrophilus* were documented by MLEE (Caugant et al., 1990), and partial sequencing of translation initiation factor 2 (*infB*) separated isolates of *Haemophilus segnis* into two discrete clusters (Hedegaard et al., 2001). Additionally, an isolate cultured from an animal bite did not belong to any described *Aggregatibacter* species by phenotype and genome sequencing (Murra et al., 2018).

Genome sequencing has become a pillar of bacterial taxonomy. Several internet services can guide species identification based on genomic sequences (Goldfarb et al., 2025; Jolley et al., 2012; Meier-Kolthoff et al., 2022; Parks et al., 2020), but classifications rarely comply with nomenclature issued by the International Committee on Systematics of Prokaryotes (ICSP). The Genome Taxonomy Database (Release 226) distributes *Aggregatibacter* Whole Genome Sequences (WGSs) from the public repositories into 12 different genomospecies (https://gtdb.ecogenomic.org/). Personal experience with unidentifiable, clinical isolates motivated us to update the taxonomy of genus *Aggregatibacter* using phenotypic and genomic analyses.

## 2 Materials and methods

### 2.1 Investigated strains and WGSs

**Figure 1:**
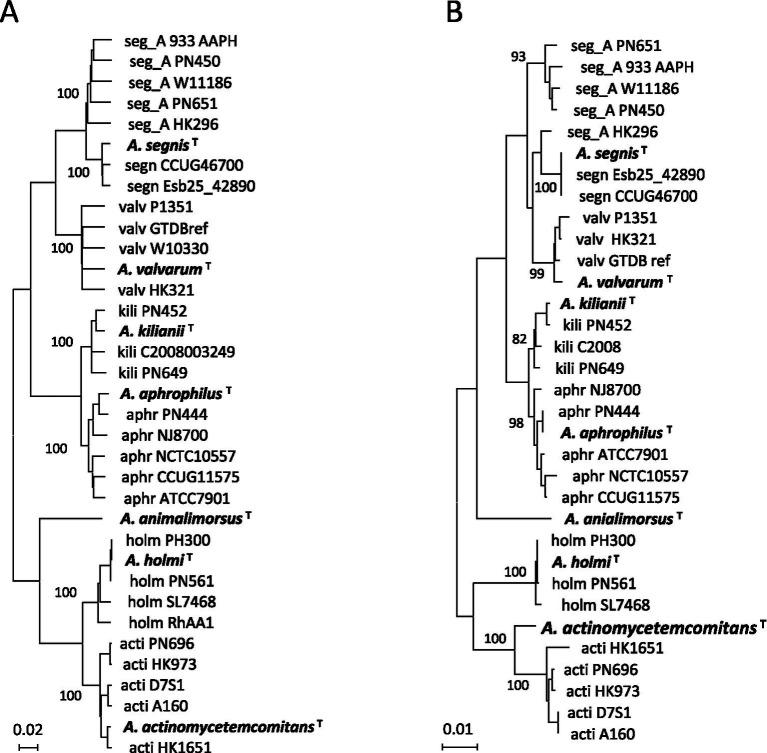
Neighbour-joining comparison of DNA from cultured *Aggregatibacter* strains. **(A)** 967 core genes (spanning 993.217 nucleotides) identified by Prokka and compared by Panaroo from 35 WGSs; pairwise SNP numbers varied from 352 (*Aggregatibacter holmi* type vs strain PH300) to 180.649 (*Aggregatibacter valvarum* GTDBref vs. *Aggregatibacter holmi* PN561). **(B)** Small subunit ribosomal ribonucleic acid (SSU or 16S rRNA) genes from 33 WGSs [1.544–1.548 nt except *Aggregatibacter aphrophilus* ATCC7901 that is 3′ truncated (1.460 nt)]. SSU RNA from *Aggregatibacter holmi* RhAA1 could not be identified in the deposited WGS, and *Aggregatibacter valvarum* W10330 was omitted because of a 480 nt 5′ truncation. Values at nodes are percentages of 500 bootstrap replications supporting the node. Bars represent 2 or 1 substitutions per 100 nucleotides, respectively.

We examined 282 WGSs from the public repositories, supplemented with recent isolates (see below). Thirty-five WGSs of eight taxa (3–6 WGSs per taxon, except suggested species *A. animalimorsus* where only 1 WGS is available) were selected for more detailed genomic characterisation (selected strains and accession numbers are listed in Supplementary Table S1). All 35 WGSs represent cultured isolates. Almost complete 16S rRNA gene sequences could be extracted from 33 of 35 WGSs, see caption to Figure 1. Fourteen sequenced strains were selected for extended phenotypic characterisation (2 isolates from each of six taxa, 1 isolate from suggested species *A. animalimorsus* and *A. valvarum*).

### 2.2 Genome taxonomy database (GTDB)

**Figure 2:**
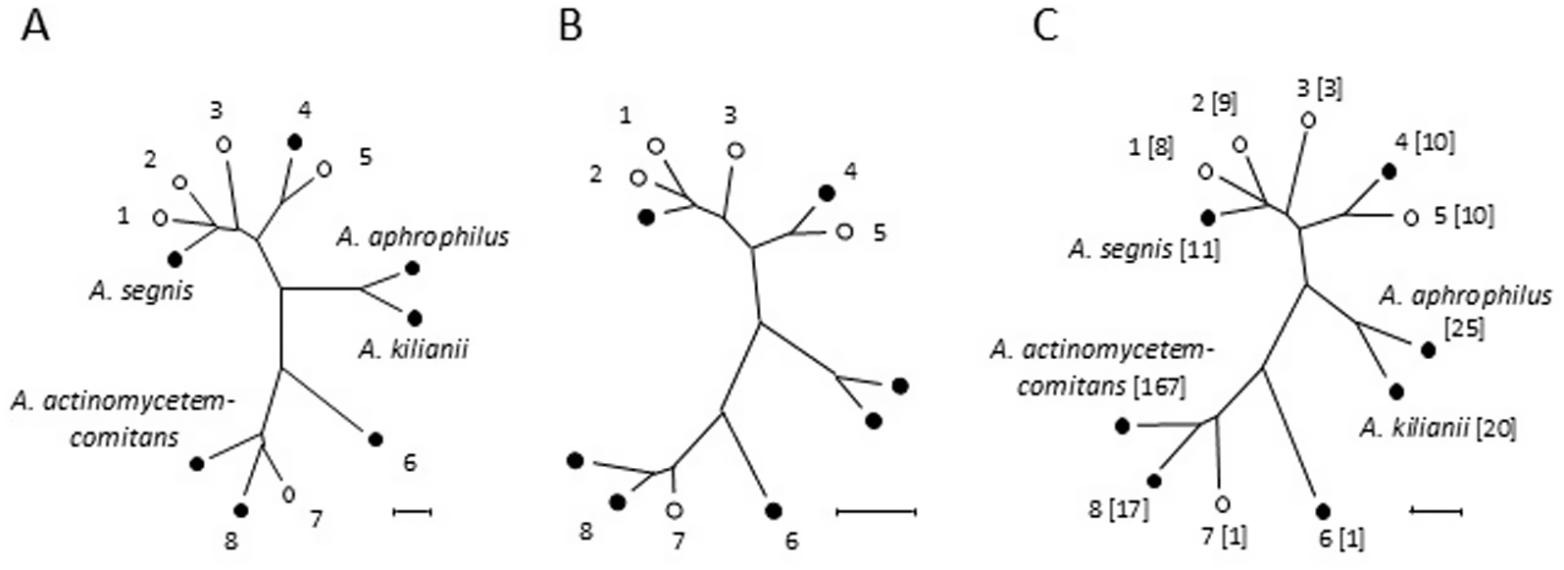
Phylogeny of genus *Aggregatibacter* as evaluated by genome analysis of type- and reference WGSs from 12 genomospecies. Filled circles represent validated or designated type strains; empty circles are GTDB representatives of genomospecies. The four validly published species names are given in **(A,C)**; unnamed genomospecies are numbered 1–8. Dendrograms are swapped to comply with analogous orientation. The number of deposited WGSs (GTDB R226) within each genomospecies is indicated in square brackets in **(C)**. Twelve WGSs are compared by: **(A)** SNP analysis of 600 core genes (583,223 nt); **(B)** FastTree analysis; **(C)** Mashtree analysis. Bars indicate branch length scale: 0.02 substitutions per site in **(A)**, 0.05 substitutions per site in **(B)**, and 0.02 Mash distance units in **(C)**.

GTDB Release 10-RS226 (16th April 2025) (https://gtdb.ecogenomic.org/) allocates 282 WGSs to genus *Aggregatibacter*; 223 WGSs belong to four validly named species, and 59 WGSs belong to 8 unnamed genomospecies. Here we examine isolates of four unnamed genomospecies, in addition to the four named species. GTDB designations of the four examined genomospecies are: s_Aggregatibacter actinomycetemcomintans_A (proposed name *Aggregatibacter holmi*, sp. nov.); s_Aggregatibacter kilianii_A (proposed name *Aggregatibacter animalimorsus*, sp. nov.); s_*Aggregatibacter segnis*_A; and s_Aggregatibacter sp000466335 (proposed name *Aggregatibacter valvarum* sp. nov.). Reference WGSs from each of the 12 GTDB taxa were used for a general outline of the genus (Figure 2); all 282 *Aggregatibacter* WGSs from the public repositories were subjected to BLAST for analysis of *nadV*.

### 2.3 Phenotypic and genomic analysis

Key phenotypic tests (including dependence on NAD, synthesis of porphyrin, activity of *β*-galactosidase (dissolution of ortho-nitrophenyl-β-galactoside, ONPG), tryptophanase (indole production), urease, and ornithine decarboxylase) were performed according to accepted guidelines (Carroll, 2023). Supplemental biochemical tests were performed with API NH and VITEK 2 NH identification cards (bioMérieux, Herlev, Denmark).

New WGSs were generated using Rapid Barcoding Kit 96 (SQK-RBK114.96), loaded onto a MinION Flow cell (R10.4.1) and sequenced on a MinION instrument using MinKNOW 25.03.9 (all from Oxford Nanopore Technologies plc, Oxford, UK). Reads were basecalled in super-accuracy mode with the dna_r10.4.1_e8.2_400bps_sup@v5.2.0 model (minimum Q-score 10), assembled with Dragonflye v1.2.1 (Flye), and polished with 1 round of Racon. Sequencing depth was 69–305 × (Supplementary Table S2). Genomes were annotated with Prokka and core genes were identified and aligned with Panaroo, as previously described (Nedergaard et al., 2019). Maximum-likelihood phylogenies were inferred from the Panaroo core-gene alignments with IQ-TREE, selecting the best-fit nucleotide-substitution model with ModelFinder and estimating branch support from 1,000 ultrafast bootstrap (UFBoot) replicates; the model selected for each tree is given in the legends to Figures 1, 2, plus Supplementary Figure S1. Some alignments were additionally compared with FastTree (GTR + CAT), an approximate maximum-likelihood method (Price et al., 2010), and genome-wide divergence was estimated with Mashtree (k-mer length 21, sketch size 10,000), which reduces nucleotide stretches of length k (k-mers) to hashed integers and counts the pairwise number of shared hashes (Katz et al., 2019). Annotation, core-gene extraction, and the FastTree and Mashtree analyses were orchestrated with compareM2 (2.16.1) (Kobel et al., 2025). BLAST analyses were performed locally with command-line BLAST+ v2.16.0. For the *nadV* screen, the *nadV* gene of the *A. aphrophilus* type strain (GenBank DQ223269.1) was used as query in `blastn` (−perc_identity 50 -evalue 1 -outfmt 6 -task blastn) against each of the 282 R226 *Aggregatibacter* genomes; genes were scored as full-length, truncated, or absent from the alignment length/identity (Supplementary Table S3 lists *nadV* status of 35 selected WGSs). 16S rRNA gene sequences were extracted from selected WGSs using BV-BRC (Olson et al., 2022), aligned with MAFFT, and compared by maximum likelihood with IQ-TREE under the model and bootstrap settings described above; full-length *nadV* genes recovered from the BLAST screen were aligned and analysed in the same way (Supplementary Figure S1C). Pairwise comparison of genomes was performed by Average Nucleotide Identity (ANI) (Yoon et al., 2017) and Genome-to-Genome Distance Calculator (GGDC) (Meier-Kolthoff et al., 2022). Sequence types of *A. holmi* were obtained by PubMLST for *A. actinomycetemcomitans* (Jolley et al., 2018).

### 2.4 Software environments

Two conda environments were created: aggre_r226 (Prokka 1.14.6, BLAST+ 2.16.0, MAFFT 7.526, Barrnap 0.9, FastTree 2.2.0) and aggre_phylo (Panaroo 1.7.0, Mashtree 1.4.6, Mash 2.3, fastANI 1.34, IQ-TREE 3.1.2, MAFFT 7.526, Barrnap 1.10.6, BLAST+ 2.17.0, seqkit 2.13.0, Biopython 1.87; R 4.5.3 with ggtree 4.0.4, treeio 1.34.0, ggplot2 4.0.3, patchwork 1.3.2, ape 5.8–1 for phylogenetic figure generation). The detailed environment information is listed under Supplementary file, conda environments (Data Sheet 2).

### 2.5 Accession numbers

Genome accession numbers of suggested type strains are GCA_980469995 (*A. animalimorsus*), GCA_980469465 (*A. holmi*), and GCA_980468985 (*A. val*var*um*). Genome accession numbers of two additional, cultured isolates of suggested species *A. valvarum* are GCA_980470455 and GCA_980470525 (HK321 and P1351, respectively). Further WGSs are deposited under project PRJEB107187. Full-length 16S rRNA gene sequence of the new type strains are deposited under accession numbers PV955042-PV955045.

## 3 Results

### 3.1 Genomic phylogeny

As defined by a 95% Average Nucleotide Identity (ANI) threshold, GTDB R226 separates 282 *Aggregatibacter* WGSs from the public repositories into 12 genomospecies, of which four carry validly published names. Figure 2 compares the type- or reference-strain genome of each of the 12 genomospecies by SNP analysis of 600 core genes (1A), by FastTree (1B), and by Mashtree (1C). Overall configuration of the dendrograms is preserved, although interspecies distances vary between methods. It is apparent that the four validly named species only represent selected positions within the genus, with *A. actinomycetemcomitans* and *A. segnis* being located on opposite edges, while *A. aphrophilus* and *A. kilinaii* take up an intermediary location. Unnamed genomospecies 1 and 2 are related to *A. segnis*, and the number of deposited WGSs are similar among the three species (11, 8, and 9, respectively, see Figure 2C). Genomospecies 7 and 8 are related to *A. actinomycetemcomitans*, but the number of deposited WGSs differs widely (1, 17, and 167, respectively).

Regarding nomenclature, genomospecies 3 and 5 are only represented by metagenome assembled genomes. Uncultured bacteria can neither be phenotypically examined, nor validly named (https://www.the-icsp.org/).

### 3.2 Phenotypic analysis

We investigated 14 strains of 8 genomospecies (Table 1). Two GTDB genomospecies have never been cultured, while two other genomospecies (#2, three of nine WGSs represent cultured isolates; #7, the single available WGS represents a cultured isolate) were not available for the present analysis. The 14 strains characterised by phenotypic analysis are also included in the extended genomic investigation (section 3.4). Phenotypic data support the genomic discrimination of Figure 2. Phosphatase is important for identification of suggested species *A. animalimorsus* (#6 in Figure 2), and for differentiation of suggested species *A. holmi* (#8) from *A. actinomycetemcomitans*. The used tests do not discriminate strains of suggested species *A. valvarum* (#4) from the *A. segnis* cluster, but mass spectrum of proteins by MALDI-TOF is clearly unique for *A. valvarum* (se Supplementary Figure S2). Moreover, three of five cultured isolates of *A. valvarum* originate from blood stream infections, which is not typical for strains of the *A. segnis* cluster. Finally, strains of s_*Aggregatibacter segnis*_A (#1) degrade carbohydrates more voraciously than strains of *A. segnis*. Similar number of WGSs are deposited for *A. segnis* and neighbouring genomospecies (Figure 2C), but s_*Aggregatibacter segnis*_A is primarily represented by cultured isolates (5 of 8 WGSs). Possibly, the metabolism of genomospecies 1 facilitates culture of these strains in the clinical microbiology laboratory.

**Table 1:** Phenotypic characterisation of 14 strains from eight *Aggregatibacter* genomospecies. Designated species names are in square brackets. Genomospecies numbers from Figure 2 are given in round brackets.

*Aggregatibacter* species	Strain	Sat^1^	FRU†	GLU†	dGLU‡	dGAL‡	SAC†	SAC‡	ßGAL†	ßGALi‡	ELLM‡	GGT†	GGT‡	PHOS‡	Pen1^2^	AMC^2^
*Aggregatibacter actinomycetemcomitans*	NCTC9710^T^	0	+	+	+	0	0	0	0	0	+	+	0	0	6	16–17
*Aggregatibacter actinomycetemcomitans*	HK 1651	0	+	+	(+)	0	0	0	0	0	+	+	0	0	13–14	14
[*Aggregatibacter holmi*] (#8)	PH791/56^T^	0	+	+	+	0	0	0	0	0	+	+	0	+	6	14–15
[*Aggregatibacter holmi*] (#8)	PN 561	0	+	+	+	0	0	(+)	0	0	+	+	0	+	6	14–16
[*Aggregatibacter animalimorsus*] (#6)	PN 491	+	+	+	+	+	+	+	(+)	+	+	+	+	0	12	18
*Aggregatibacter aphrophilus*	ATCC33389^T^	0	+	+	+	+	+	+	+	+	+	+	0	+	17	20–24
*Aggregatibacter aphrophilus*	HK 415	+	+	+	+	+	+	+	+	+	+	+	+	+	14	18
*Aggregatibacter kilianii*	PN 452	+	+	+	+	+	+	+	+	+	+	+	0	+	18–20	16
*Aggregatibacter kilianii*	CCUG70536^T^	0	+	+	+	0	+	+	(+)	+	(+)	(+)	+	+	14	20
[*Aggregatibacter valvarum*] (#4)	Herl E20^T^	+	(+)	+	0	0	+	0	0	0	0	0	0	+	18	18
*Aggregatibacter segnis*	ATCC33393^T^	+	w	w	0	0	w	w	0	0	0	0	0	+	12	18
*Aggregatibacter segnis*	CCUG46700	+	0	+	0	0	0	0	0	0	0	0	0	+	11	18–20
s_*Aggregatibacter segnis*_A (#1)	HK 296	+	+	+	0	0	+	0	0	0	0	0	0	+	18	20
s_*Aggregatibacter segnis*_A (#1)	PN_450	+	+	+	0	0	+	0	0	0	0	0	0	+	18	18

+, positive; 0, negative; (+), weak reaction.

^1^Satellitism (NAD-dependence); on 5% horse blood agar, colonies are only visible adjacent to breeder colonies (eg *Staphylococcus aureus* ATCC 29213).

†Analysed with API-NH.

‡Analysed with VITEK-NH.

^2^Inhibition zone (mm) circumscribing benzylpenicillin (1 unit) and amoxicillin-clavulanate (2–1 μg) disks on MH-F agar plates according to EUCAST; range of three experiments.

The two last columns of Table 1 list disk-diffusion susceptibility testing of benzylpenicillin and amoxicillin-clavulanate. EUCAST (v15.0) does not provide interpretative criteria for *Aggregatibacter*; for *Haemophilus influenzae* and *Pasteurella* species, susceptibility to penicillins is inferred from the benzylpenicillin 1 unit disk diffusion screening test, while interpretation of amoxicillin-clavulanate susceptibility is restricted to ß-lactamase-positive isolates (https://www.eucast.org). All strains of Table 2 were negative for ß-galactosidase (nitrocefin test) (not shown), therefore, amoxicillin-clavulanate zones represent susceptibility to aminopenicillins. The benzylpenicillin screening breakpoints may not be useful for interpretation of *Aggregatibacter* susceptibility, since four strains exhibit inhibition zones below the breakpoint for *H. influenzae* (R < 12 mm), and five additional strains have inhibition zones below the breakpoint for *Pasteurella* (R < 17 mm) (https://www.eucast.org). Only *A. actinomycetemcomitans* strain HK1651 is resistant to oral amoxicillin-clavulanate (equal breakpoints (R < 15 mm) are defined for *H. influenzae* and *Pasteurella*), but resistance to amoxicillin in *A. actinomycetemcomitans* has not been identified using state of the art agar dilution method (Jensen et al., 2019).

**Table 2:** DNA comparison of *Aggregatibacter* type strains plus GTDB reference strain HK296. Proposed species names are in sharp brackets.

Species (number in Figure 2)	Strain	NCTC9710^T^	PH791/56^T^	PN491^T^	ATCC33389^T^	PN528^T^	HerlE20^T^	ATCC33393^T^	HK296
*Aggregatibacter actinomycetemcomitans*	NCTC9710^T^	-	**93.77**	86.09	81.79	81.39	81.29	81.81	81.70
[*Aggregatibacter holmi*] (#8)	PH791/56^T^	**54.50**	-	86.04	81.70	81.49	81.67	81.74	81.37
[*Aggregatibacter animalimorsus*] (#6)	PN491^T^	30.80	30.70	-	81.99	81.83	82.03	82.56	82.57
*Aggregatibacter aphrophilus*	ATCC33389^T^	24.70	24.70	24.40	-	**94.00**	85.56	84.52	84.53
*Aggregatibacter kilianii*	PN528^T^	24.20	24.30	24.50	**54.30**	-	85.92	85.07	85.06
[*Aggregatibacter valvarum*] (#4)	HerlE20^T^	24.70	24.60	25.40	30.80	31.40	-	89.27	88.60
*Aggregatibacter segnis*	ATCC33393^T^	24.20	24.30	25.20	28.10	29.00	39.60	-	**94.82**
s_*Aggregatibacter segnis*_A (#1)	HK296	24.20	24.40	25.10	28.20	28.90	38.70	**59.00**	-

Upper right, average nucleotide identity [https://www.ezbiocloud.net/ (Yoon et al., 2017)]. Lower left, digital DNA hybridization [https://ggdc.dsmz.de/ (Meier-Kolthoff et al., 2022)]. ANI above 90%, and dDNA distances above 50% are in bold.

### 3.3 Distribution of *nadV* in genus *Aggregatibacter*

Strains of *A. actinomycetemcomitans* possess a functional nicotinamide phosphoribosyltransferase (encoded by *nadV*). A functional gene is common, but not ubiquitous in *A. aphrophilus* and *A. kilianii*, while it has never been deteced in *A. segnis*. *nadV* has previously been characterised in *A. aphrophilus* and *A. kilianii*. With respect to *A. aphrophilus*, nine of 10 NAD-dependent isolates carried a short, truncated version of *nadV*. The last NAD-dependent strain (CCUG 41544) carried a single nucleotide deletion in *nadV* that gave rise to a reading-frame shift (Nørskov-Lauritsen and Kilian, 2006). Ten isolates of *A. kilianii* were examined in the effective publication of that species, and a single strain (PN_452) exhibited satellitism. The NAD-dependence was caused by a single nucleotide deletion in *nadV*, where a 6-nucleotide homopolymer was terminated after 5 deoxythymidine residues (Murra et al., 2018).

Single nucleotide deletions may erroneously be identified by sequencing errors, which makes phenotypic conclusions based on WGSs fallacious. However, BLAST search of >1 kB *nadV* homologs among 282 *Aggregatibacter* WGSs in GTDB R226 showed full, or almost complete *nadV* genes in all genomes of *A. actinomycetemcomitans* and related genomospecies #7 and #8, in 29 of 45 genomes of *A. aphrophilus* and *A. kilianii* (56 and 75% of deposited WGSs, respectively), but in none of 52 WGSs representing *A. segnis* and the six remaining genomospecies. Thus, bioinformatics analysis of deposited WGSs indicate that – except for the two genomospecies closely related to *A. actinomycetemcomitans* - all unnamed genomospecies of *Aggregatibacter* will express satellitism when cultured on 5% blood agar, due to the absence of *nadV*.

*nadV* is located between *thrA* (bifunctional aspartokinase/homoserine dehydrogenase gene) and *yggS* (pyridoxal phosphate homeostasis protein gene) in *Pasteurellaceae*, and the genomic region was examined in the 35 WGSs included in Figure 1. The distance between *thrA* and *yggS* spanned 1733–1752 nt in *A. actinomycetemcomitans*, *A. holmi*, and *A. kilianii* (including PN_452 with a non-functional NadV), and in three of six WGSs of *A. aphrophilus*. In contrast, only 209–10 noncoding nt separated *thrA* and *yggS* in all 14 WGSs of *A. segnis*, s_*Aggregatibacter segnis*_A, *A. valvarum*, and *A. animalimorsus*. The distance between *thrA* and *yggS* spanned 489–495 nt in three V-factor dependent strains of *A. aphrophilus* (ATCC7901, CCUG11575 and NCTC10557), encoding short, truncated versions of *nadV* (*nadV* alignments are presented in Supplementary Figure S3). Phylogenetic comparison of the 18 full-length *nadV* genes recovered across the 35 WGSs (1389 nt; 1388 nt for strain PN_452) demonstrated a close relationship of *nadV* between *A. actinomycetemcomitans* and *A. holmi*, and between *A. aphrophilus* and *A. kilianii*. The *nadV* similarity within the two species pairs was considerably more pronounced than the similarity observed with genome-wide comparisons (Supplementary Figure S1C *vs* Figure 1A).

### 3.4 Extended genomic comparison of genomospecies

Table 2 shows pairwise DNA sequence comparison of type- or representative-WGSs of four named species plus four genomospecies (numbered 1, 4, 6 and 8 in Figure 2), as calculated by ANI and by digital DNA (dDNA) hybridisation. All WGSs qualify for separate species designation according to accepted genomic breakpoints (dDNA: 70% (Wayne, 1988); ANI: 95% (Konstantinidis and Tiedje, 2005)). Three pairs of genomospecies are closely related (*A. actinomycetemcomitans* vs. suggested species *A. holmi*, *A. aphrophilus* vs. *A kilianii*, and *A. segnis* vs. s_*Aggregatibacter segnis*_A); the distances between these pairs are highlighted. Both bioinformatics methods identify *A. segnis* and s_*Aggregatibacter segnis*_A as the most closely related pair.

Figure 1A shows SNP analysis of 967 core genes identified in 35 *Aggregatibacter* WGSs. The core genome (number of core genes) is larger than that of Figure 2, because the analysis is reduced from 12 to 8 genomospecies. Core gene comparison separates 35 WGSs into eight species clusters, all supported by a bootstrap value of 100%. Suggested species *A. valvarum* and *A. animalimorsus* are clearly distinct entities, while s_*Aggregatibacter segnis*_A is close to *A. segnis*, *A kilianii* is close to *A. aphrophilus*, and suggested species *A. holmi* is close to *A. actinomycetemcomitans*. Compared with s_*Aggregatibacter segnis*_A, the genomes of *A. segnis* appear more homogenous. The comparison of WGSs is restricted to cultured isolates, and only few representatives of *A. segnis* are available in the public repositories. Inclusion of a recently cultured strain (Esb25_42890) did not significantly expand the genomic heterogeneity of *A. segnis* (Figure 1A, top). Although the comparison is restricted to a limited number of sequences, it cannot be excluded that the voracious metabolism of s_*Aggregatibacter segnis*_A supports identification of more diverse subtypes by routine culture in the clinical laboratory. In addition to the two WGSs of cultured strains of suggested species *A. valvarum* available in the public repositories (blood sample strain GCA_017798005.1 and oral strain GCA_000466335.1), we have deposited the WGS of strain HerlE20 from a case of infective endocarditis (suggested type strain), plus two WGSs from the cryptic cluster of *A. segnis* identified many years ago by partial sequencing of *infB* (Hedegaard et al., 2001) (HK321 and P1351 from dental plaque and blood, respectively, see Supplementary Table S1).

For decades, 16S rRNA has been pivotal for categorisation of prokaryotes. Figure 1B compares full-length 16S rRNA gene sequences identified in 33 *Aggregatibacter* WGSs (see caption for missing sequences). 16S rRNA comparison supports delineation of most *Aggregatibacter* genomospecies, albeit the bootstrap support of nodes is reduced. Moreover, conspicuous dissimilarities are visible. The 16S rRNA gene of GTDB reference strain of s_*Aggregatibacter segnis*_A (HK296) clusters with *A. segnis*, in contrast to the position when core genes are compared (Figure 1A). By 16S rRNA, suggested species *A. holmi* is widely separated from *A. actinomycetemcomitans*, confirming the peculiar 16S rRNA type V sequence defined by Kaplan et al. (2002). Finally, the 16S rRNA gene of the type strain of *A. actinomycetemcomitans* (NCTC9710^T^) is not typical of this species.

## 4 Discussion

*Aggregatibacter* species are human commensals (Nørskov-Lauritsen et al., 2019), but sporadic culture from animals or animal bites suggests a wider host range. The oral cavity is the principal location (Haubek et al., 2008), although deposited metagenome-assembled genomes of *A. segnis* and some other genomospecies frequently originates from faeces. A conspicuous association of certain species with particular infections is documented, such as *A. aphrophilus* and brain abscess (Kommedal et al., 2014); long before the recognition of the species, strain ATCC7901 (Pitman 429, formerly identified as *Haemophilus parainfluenzae*) was cultured from the spinal fluid of a child with brain abscess (Pittman, 1940). But nomenclature of the genus is incomplete, as documented in this study.

We investigated four unnamed genomospecies of genus *Aggregatibacter*. A single strain (PN491^T^) isolated from a human wound after an animal bite is unique (#6 in Figure 2), as there are no closely related WGS in the public repositories. It can be argued that a single strain is insufficient for naming of new species (Christensen et al., 2001); however, the exceptional position of the WGS justifies a validly published name for overall orientation of the genus in future investigations. We propose *Aggregatibacter animalimorsus* sp. nov. for this species.

The so-called clade e’ subtype of *A. actinomycetemcomitans* was recognised in 1994 (Poulsen et al., 1994). We confirm the distinctiveness of the genomospecies by phenotype and genomic analysis [#8 in Figure 2] and propose *Aggregatibacter holmi* sp. nov. for this genomospecies. Approximately 10% of WGSs deposited as *A. actinomycetemcomitans* in the public repositories are actually *A. holmi*. Valid publication of *A. holmi* will enable elucidation of the clinical significance of this species.

We propose *Aggregatibacter valvarum* sp. nov. for genomospecies #4 in Figure 2 (GTDB R226 designation: s_Aggregatibacter sp000466335). *A. valvarum* is clearly distinct by WGS and MALDI-TOF analysis; moreover, it is conspicuous that three of five cultured isolates originate from blood stream infections. The specific epithet reflects this correlation.

s_*Aggregatibacter segnis*_A (genomospecies 1 in Figure 2) can aspire for nomenclatural recognition according to phenotype (Table 1), by comparison of WGSs (Figure 1A), and to some extent by comparison of 16S rRNA (Figure 1B). The metabolism of s_*Aggregatibacter segnis*_A is remarkable. However, bioinformatics methods document that *A. segnis* and s_*Aggregatibacter segnis*_A are closely related, and there are no clinical data to support their discrimination. Currently, we abstain from specific validation of s_*Aggregatibacter segnis*_A.

Dependence on exogenous growth factors has been decisive for classification of *Pasteurellaceae*, particularly *Haemophilus*. Genus *Aggregatibacter* is unusual as it encompasses species that are dependent, and species that are independent on “V-factor” (NAD), which is molecularly defined by absence or presence of a functional nicotinamide phosphoribosyltransferase. Broad access to genome sequencing has reduced the taxonomic importance of this trait but concurrently allowed for elucidation of the characteristic within the genus. Single nucleotide deletions in *nadV* may give rise to exceptional strains that oppose the common phenotype of a NAD-independent species, but bioinformatics analysis of available *Aggregatibacter* WGSs shows that a complete *nadV* gene is ubiquitous in *A. actinomycetemcomitans*, *A. holmi* and the single published WGS of the closely related genomospecies 7. Apart from these related taxa, *Aggregatibacter* strains and genomospecies do not harbour *nadV*, or truncated versions of the gene. The exception to this principle is the two related species *A. aphrophilus* and *A. kilianii*. Merging of former species *Haemophilus aphrophilus* and *Haemophilus paraphrophilus* into one taxon reflects this perception, and current bioinformatics analysis of 45 genomes of *A. aphrophilus* and *A. kilianii* show that *nadV* is dispensable in these two species, although fragments of the gene are usually present. Whether a functional nicotinamide phosphoribosyltransferase is important for certain habitats and infections of just these two *Aggregatibacter* species is currently unknown.

Images of colony morphology after 20 and 44 h on chocolate agar plates of seven *Aggregatibacter* type strains are included in Supplemented Figure S4.

### 4.1 Description of *Aggregatibacter animalimorsus* sp. nov.

*Aggregatibacter animalimorsus* (a.ni.ma.li.mor’sus. L. neut. n. *animal*, an animal; L. gen. Masc. n. *morsus*, a bite; N. L. gen. Masc. n. *animalimorsus*, of an animal bite, as the type strain was isolated from a localized human infection after an animal bite). Gram-negative, nonmotile, facultatively anaerobic, short, regular rods (0.5 by 1.6 μm). Colonies on chocolate agar incubated in air supplemented with 5% CO_2_ are convex, granular, and opaque and reach a diameter of 1.0 to 1.5 mm within 24 h. Porphyrins are synthesized from d-aminolaevulinic acid (X factor is not required); the type strain does not encode *nadV*, and exogenous NAD (V factor) is required. Results are negative in the three tests used for biotyping of *H. influenzae* and *H. parainfluenzae* (indole, urease, and ornithine decarboxylase).

Acid is produced from glucose, fructose, maltose, mannose, sucrose, and ribose, while galactose, maltotriose, and xylose are not fermented. Ortho-Nitrophenyl-*β*-galactoside (ONPG) is hydrolyzed; positive reactions with Ellman’s reagent (concentration of thiol groups, ELLM) and gamma-glutamyltransferase (GGT); negative for hydrolysis of phosphoric acid (PHOS). Key phenotypic tests for discrimination between *Aggregatibacter animalimorsus* and the other species of genus *Aggregatibacter* are fermentation of glucose, and detection of ELLM, GGT, PHOS, and ß-galactosidase.

*Aggregatibacter animalimorsus* was cultured from a human wound after an animal bite; the animal species was not recorded. GenBank/EMBL/DDBJ accession number of type strain genome is GCA_980469995 (Nanopore) and GCA_003129965.1 (Illumina); the 16S rRNA gene accession number is PV955042. The G + C content of the DNA of the type strain is 45.54 mol%. The type strain PN_491^T^ (=CCUG 78349^T^ = DSM 120368^T^) was isolated in Denmark in 2004.

### 4.2 Description of *Aggregatibacter holmi* sp. nov.

*Aggregatibacter holmi* (holm’i. N.L. gen. Masc. n. *holmi* of Holm, named in honour of Per Holm for his studies of actinomycosis and the isolation of the type strain in 1956). Gram-negative, nonmotile, facultatively anaerobic, short, regular rods (0.5 by 1.5 μm), which may appear as cocci in broth. Grows poorly in ambient air, but well in 5% CO_2_. Colonies on chocolate agar are small, with a diameter of 0.5 mm after 24 h, but may exceed 1–2 mm after 48 h. On primary isolation, the colonies are rough textured and adherent. Cells from rough colonies grow in broth as granular, autoaggregated cells that adhere to the glass and leave a clear broth. Successive rounds of subculturing on solid media can result in transformation into a smooth, non-adherent colony type that exhibits planktonic growth in broth. Haem and exogenous NAD (X and V factors) are not required. Results are negative for indole, urease, and ornithine decarboxylase.

Acid is produced from glucose, fructose and mannose, whereas galactose, ribose, sucrose and xylose are not fermented. Catalase-positive; *β*-galactosidase negative (ONPG is not hydrolysed). Human strains express *Aggregatibacter actinomycetemcomitans* serotype e antigen, and were previously designated as clade e’; the Rhesus macaque strain RHAA1 is serotype b. *Aggregatibacter holmi* is discriminated from *Aggregatibacter actinomycetemcomitans* by genome sequencing, hydrolysis of phosphoric acid, and fermentation of starch and glycogen. Key phenotypic tests for discrimination between *Aggregatibacter holmi* and the other species of genus *Aggregatibacter* are fermentation of glucose, and detection of ELLM, GGT, PHOS, and ß-galactosidase.

Indigenous to man, with primary habitat on dental surfaces. *Aggregatibacter holmi* has been isolated together with *Actinomyces* from human actinomycosis. Strain PN561 was cultured from a bloodstream infection, and strain RHAA1 is from *Macaca mulatta*, a species of Old-World monkey. GenBank/EMBL/DDBJ accession number of type strain genome is GCA_980469465.1 (Nanopore) and GCF_003130205.1 (Illumina); the 16S rRNA gene accession number is PV955044. The DNA G + C content of the type strain is 44.52%. The type strain PH_791/56^T^ (= HK961^T^ = CCUG 78347^T^ = DSM 120099^T^) was isolated from a human abscess in Denmark in 1956.

### 4.3 Description of *Aggregatibacter valvarum* sp. nov.

*Aggregatibacter valvarum* (val.va’rum. L. fem. n. *valva*, a folding-door, referring to the heart valve; L. gen. Fem. pl. n. *valvarum*, of heart valves). Gram-negative, nonmotile, facultatively anaerobic, short, regular rods (0.5 by 1.5 μm), with occasional filamentous forms. Colonies on chocolate agar incubated in air supplemented with CO_2_ are convex and granular, and reach a diameter of 1.0 to 1.5 mm within 24 h. Growth in broth may be granular. Porphyrins are synthesized from d-aminolaevulinic acid (X factor is not required); nicotinamide phosphoribosyltransferase is absent, and exogenous NAD (V factor) is required. Results are negative for indole, urease, and ornithine decarboxylase.

Acid is produced from glucose and sucrose, while galactose, ribose, and xylose are not fermented. Negative for β-galactosidase (ONPG), ELLM and GGT; phosphoric acid is hydrolysed. Key tests for discrimination between *Aggregatibacter valvarum* and the other species of genus *Aggregatibacter* are fermentation of glucose, and detection of ELLM, GGT, PHOS, and ß-galactosidase.

*Aggregatibacter valvarum* has only been cultured from humans, thrice from bloodstream infections, while strains W10330 and HK321 are from the oral cavity; additional metagenome-assembled oral genomes indicate that the oral cavity is the primary habitat of the species. GenBank/EMBL/DDBJ accession number of type strain genome is GCA_980468985.1; the 16S rRNA gene accession number is PV955045. The DNA G + C content of the type strain is 43.34%. The type strain HerlE20^T^ (=CCUG 78345^T^ = DSM 120369^T^) was isolated from a case of infective endocarditis in Denmark in 2020.

## Data Availability

The datasets presented in this study can be found in online repositories. The names of the repository/repositories and accession number(s) can be found in the article/Supplementary material.
