# Supplementary material for: Distribution of nicotinamide phosphoribosyltransferase in genus Aggregatibacter and description of Aggregatibacter animalimorsus sp. nov., Aggregatibacter holmi sp. nov., and Aggregatibacter valvarum sp. nov
Source: Front Microbiol. 2026 Jul 29;17:1866808. doi: 10.3389/fmicb.2026.1866808 (PMC13461592; doi:10.3389/fmicb.2026.1866808)
Supplement: Supplementary file 1 [file Data_Sheet_1.PDF]

List of Supplemental Tables and Figures

FILE #	FILE	PAGE
1	Supplemental Table S1. List of strains for genome analysis	2
2	Supplemental Table S2. Genome sequence statistics	4
3	Supplemental Table S3. *nadV* status of GSs included in Figure 2	5
4	Supplemental Figure S1. Comparative IQ TREEs including *nadV*	6
5	Supplemental Figure S2. Mass spectrometry of *Aggegatibacter*	7
6	Supplemental Figure S3. nadV sequence alignment	10
7	Supplemental Figure S4. Colony appearance	15

Supplemental Table S1. List of strains for genome analysis

Species	Strain	Type	Subtype	Source	Origin	Phenotype (Table 1)	Other Designation	Sequence #
A. actinomycetemcomitans	NCTC9710^{T}	yes	cluster I, St a	Abscess	Denmark 1955	yes	CCUG13227^{T}	GCF_008085305.1
A. actinomycetemcomitans	HK1651	-	cluster I, St b	Subgingival plaque	Denmark 1995	yes	CCUG56173	GCF_000604045.1
A. actinomycetemcomitans	D7S1	-	cluster II, St a	Periodontitis	USA before 2002	-		GCF_000163615.3
A. actinomycetemcomitans	A160	-	cluster II, St e	Periodontitis	Finland 1995	-		GCF_000332915.2
A. actinomycetemcomitans	HK973	-	cluster III, St a	Subgingival plaque	Denmark 1991	-		GCF_008086385.1
A. actinomycetemcomitans	PN696	-	cluster III, St a	Blood	Denmark 2010	-	B10-001802	GCF_008086405.1
A. animalimorsus	PN491^{T}	yes	-	Animal bite	Denmark 2004	yes	HerP04-240792	GCA_980469995.1
A. aphrophilus	ATCC33389^{T}	yes	-	Endocarditis	England 1939	yes	HK372^{T}	GCF_000226495.2
A. aphrophilus	ATCC7901	-	NAD-dep	Brain abscess	USA 1932	-	Pittman 429	GCF_001680805.1
A. aphrophilus	CCUG11575	-	NAD-dep	Brain abscess	Sweden 1981	-		GCF_003130375.1
A. aphrophilus	NCTC10557	-	NAD-dep	Paronychia	England 1968	yes	HK415	GCA_982384155.1
A. aphrophilus	NJ8700	-	-	Oral cavity	US, before 2009	-		GCF_000022985.1
A. aphrophilus	PN444	-	-	Brain abscess	Denmark 2008	-	Aal08-40496	GCF_003130145.1
A. holmi	PH791/56^{T}	yes	(Aa) ST 57	Abscess	Denmark 1956	yes	HK961^{T}	GCA_980469465.1
A. holmi	PH300	-	(Aa) ST 11	Abscess	Denmark 1961	-	HK964	GCF_021309605.1
A. holmi	PN561	-	(Aa) ST 12	Blood	Denmark 2005	yes	Her05-6229	GCF_008085015.1
A. holmi	RhAA1	-	St b	(Macaca mulatta)	USA, before 2011	-		GCF_000259915.1
A. holmi	SL_7468	-	St f	(Macaca mulatta)	USA 2010	-		GCF_017160935.1
A. kilianii	PN528^{T}	yes	-	Abdominal abscess	Denmark 2009	yes	CCUG70536^{T}	GCF_003130255.1
A. kilianii	C2008003249	-	-	Sinus	USA 2008	-		GCA_003252995.1
A. kilianii	PN452	-	NAD-dep	Conjunctivitis	Denmark 2012	yes	Aal12-P/014757	GCF_003130055.1
A. kilianii	PN649	-	-	Blood	Denmark 2014	-	Ode14-925921	GCF_003130015.1
A. segnis	ATCC 33393^{T}	yes	-	Dental plaque	Denmark 1975	yes	HK316^{T}	GCF_000185305.1
A. segnis	CCUG46700	-	-	Abscess	Sweden 2002	yes		GCF_003130095.1
A. segnis	Esb25	-	-	Conjunctivitis	Denmark 2025	-	A42890	GCA_978484445.1
*A. segnis*_A	HK296	(ref)	-	Sputum	Denmark 1973	yes	DSM 120370	GCF_003130075.1
*A. segnis*_A	933_AAPH	-		n/a	USA, before 2015	-		GCF_001059425.1

*A. segnis*_A	PN450	-	-	Blood	Denmark 2000	yes	Aal00-106911	GCF_003130345.1
*A. segnis*_A	PN651	-	-	Blood	Denmark 2013	-	Ode13-325455	GCF_003130325.1
*A. segnis*_A	W11186	-	-	Oral cavity	UK, before 2021	-		GCF_018128305.1
A. valvarum	HerlE20^{T}	yes	-	Endocarditis	Denmark 2020	yes	DSM120369^{T}	GCA_980468985.1
A. valvarum	(GTDBref)	-	-	Blood	Australia 2021	-		GCF_017798005.1
A. valvarum	W10330	-	-	Oral cavity	USA, before 2014	-		GCF_000466335.1
A. valvarum	HK321	-	-	Dental plaque	Denmark 1975	-		GCA_980470455.1
A. valvarum	P1351	-	-	Blood	Denmark 1991	-	CCUG51799	GCA_980470525.1

Supplemental Table S2. Genome sequence stats

Strain	*Species*	*ENA_sample*	*GCA*	*Culture_collection*	*Type_strain*	*Depth_x*	*Contigs*	*Genome_size_bp*	*N50_bp*	*GC_%*	*CDS*
PN491	A. animalimorsus	ERS29095029	GCA_980469995	CCUG 78349, DSM 120368	yes	88	2	2742089	2733061	45.54	2545
HK961	A. holmi	ERS29095030	GCA_980469465	CCUG 78347, DSM 120099	yes	170	1	2250664	2250664	44.52	2163
PN561	A. holmi	ERS29095031		CCUG 78350, DSM 120398	no	109	2	2237540	2213195	44.46	2139
HK296	A. segnis	ERS29095032		CCUG 78346, DSM 120370	yes	89	1	2007643	2007643	42.71	1849
PN450	A. segnis	ERS29095033		CCUG 78348, DSM 120123	no	121	1	1964527	1964527	42.84	1829
Esb25890-3	A. segnis	ERS29095034			no	69	1	1863859	1863859	42.82	1704
HerIE20	A. valvarum	ERS29095035	GCA_980468985	CCUG 78345, DSM 120369	yes	87	1	2030755	2030755	42.9	1845
P1351	A. valvarum	ERS29095036	GCA_980470525	CCUG 51799	no	172	2	2105539	2058744	43.06	2019
HK321	A. valvarum	ERS29095037	GCA_980470455	CCUG 49495	no	305	1	2037873	2037873	42.89	1907
HK1802	A. segnis	ERS28655506		CCUG 51798	no	185	1	2020908	2020908	42.68	1885
Esb42890A1	A. segnis	ERS28655507			no	91	5	2125840	2077505	42.67	2034
HK415	A. aphrophilus	ERS29604999			no	133	1	2461944	2461944	42.3	2224

Supplemental Table S3. *nadV* status of 35 WGSs included in Figure 2

species	strain	accession	*nadV*	best_contig	span_bp
A. actinomycetemcomitans	NCTC9710	GCF_008085305.1	full	NZ_VSDU01000012.1	1389
A. actinomycetemcomitans	HK1651	GCF_000604045.1	full	NZ_CP007502.1	1389
A. actinomycetemcomitans	D7S1	GCF_000163615.3	full	CP003496.2	1389
A. actinomycetemcomitans	A160	GCF_000332915.2	full	NZ_AJME02000018.1	1389
A. actinomycetemcomitans	HK973	GCF_008086385.1	full	NZ_CP043004.1	1389
A. actinomycetemcomitans	PN696	GCF_008086405.1	full	NZ_CP043005.1	1389
A. animalimorsus	PN491	GCA_980469995.1	absent		0
A. aphrophilus	ATCC33389	GCF_000226495.2	full	NZ_AEWB02000016.1	1389
A. aphrophilus	ATCC7901	GCF_001680805.1	truncated	NZ_MAQE01000001.1	161
A. aphrophilus	CCUG11575	GCF_003130375.1	truncated	NZ_NRDC01000002.1	161
A. aphrophilus	NCTC10557	GCA_982384155.1	truncated	contig00001	91
A. aphrophilus	NJ8700	GCF_000022985.1	full	NC_012913.1	1389
A. aphrophilus	PN444	GCF_003130145.1	full	NZ_NRDA01000002.1	1389
A. holmi	PH791_56	GCA_980469465.1	full	contig00001	1389
A. holmi	PH300	GCF_021309605.1	full	NZ_JABJYQ010000014.1	1389
A. holmi	PN561	GCF_008085015.1	full	NZ_VSEC01000001.1	1389
A. holmi	RhAA1	GCF_000259915.1	full	NZ_AHGR01000002.1	1389
A. holmi	SL_7468	GCF_017160935.1	full	NZ_JACAWH010000085.1	1389
A. kilianii	PN528	GCF_003130255.1	full	NZ_NRCN01000001.1	1389
A. kilianii	C2008003249	GCA_003252995.1	full	QEPY01000003.1	1389
A. kilianii	PN452	GCF_003130055.1	full	NZ_NRCQ01000011.1	1388
A. kilianii	PN649	GCF_003130015.1	full	NZ_NRCL01000003.1	1389
A. segnis	ATCC33393	GCF_000185305.1	absent		0
A. segnis	CCUG46700	GCF_003130095.1	absent		0
A. segnis	Esb25	GCA_978484445.1	absent		0
A. segnis_A	HK296	GCF_003130075.1	absent		0
A. segnis_A	933_AAPH	GCF_001059425.1	absent		0
A. segnis_A	PN450	GCF_003130345.1	absent		0
A. segnis_A	PN651	GCF_003130325.1	absent		0
A. segnis_A	W11186	GCF_018128305.1	absent		0
A. valvarum	HerlE20	GCA_980468985.1	absent		0
A. valvarum	GTDBref	GCF_017798005.1	absent		0
A. valvarum	W10330	GCF_000466335.1	absent		0
A. valvarum	HK321	GCA_980470455.1	absent		0
A. valvarum	P1351	GCA_980470525.1	absent		0

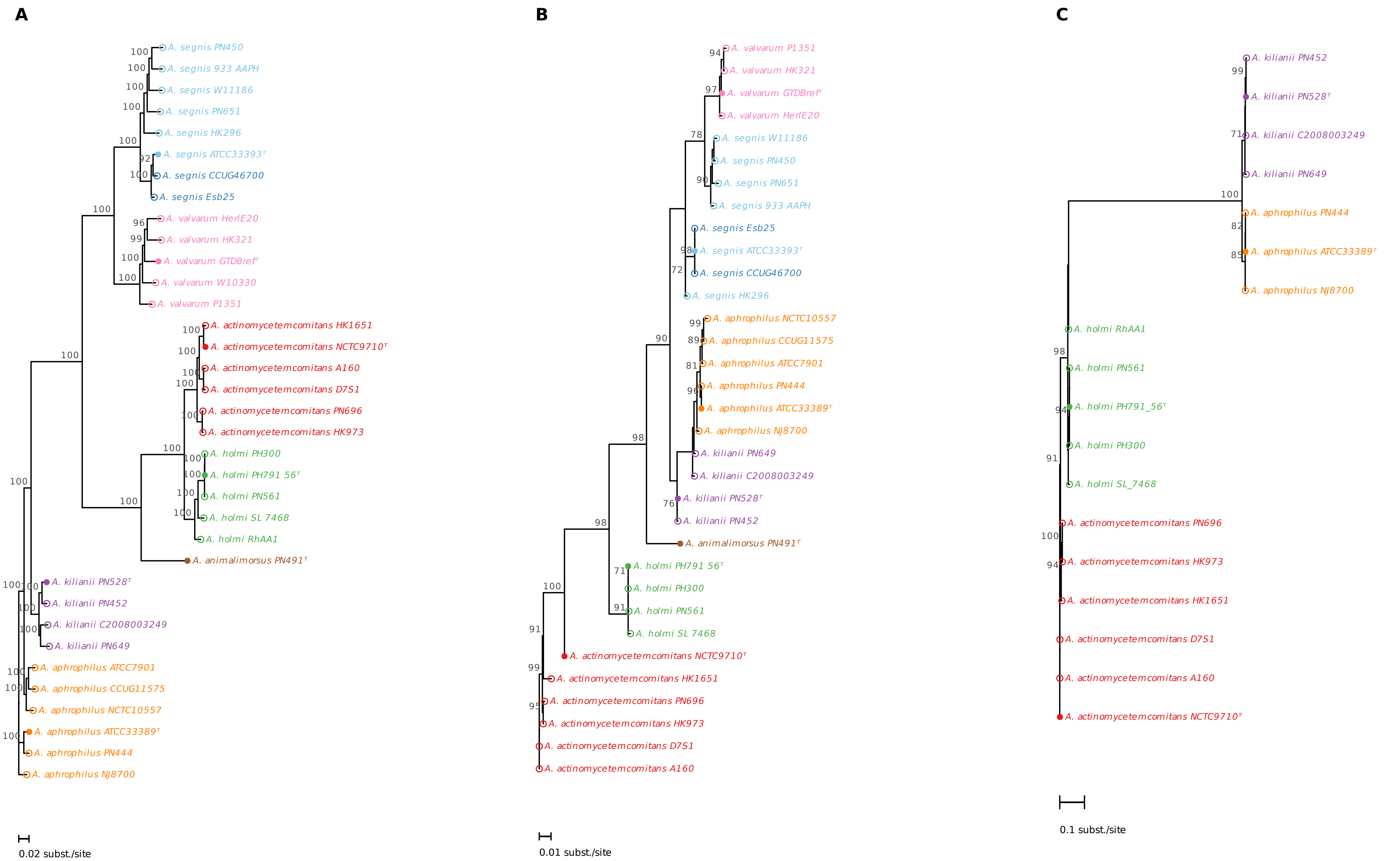

Supplementary Figure 1. Phylogenetic comparison of cultured Aggregatibacter strains.
Filled circles (●): type strains. Open circles (○): non-type strains. Bootstrap support values ≥70 shown at nodes. Scale bars represent substitutions per site.
A IQ-TREE (GTR+F+I+R6, 959 core genes, 1 026 257 nt, 35 WGSs, UFBoot 1000). B 16S rRNA IQ-TREE (TPM3u+F+I+G4, 33 WGSs, UFBoot 1000). C nadV gene IQ-TREE (codon-position-partitioned TPM3u+F+G4, 18 full-length sequences, UFBoot 1000).

Supplemental Figure S2. Mass spectrometry of *Aggegatibacter* strains

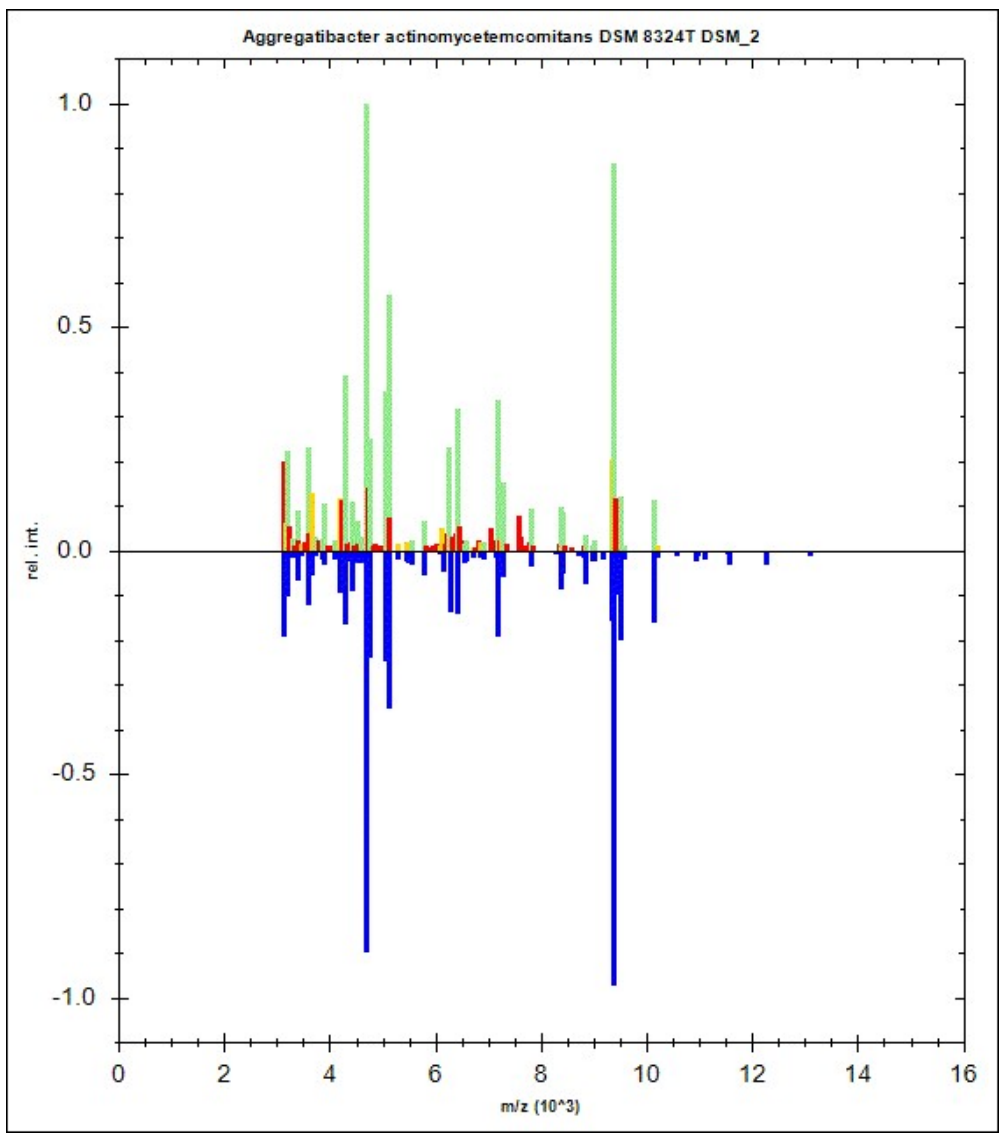

A. actinomycetemcomitans NCTC9710^{T} *vs* Bruker MSP (*A. actinomycetemcomitans*) DSM 8324T DSM_2

Log score value 2.37

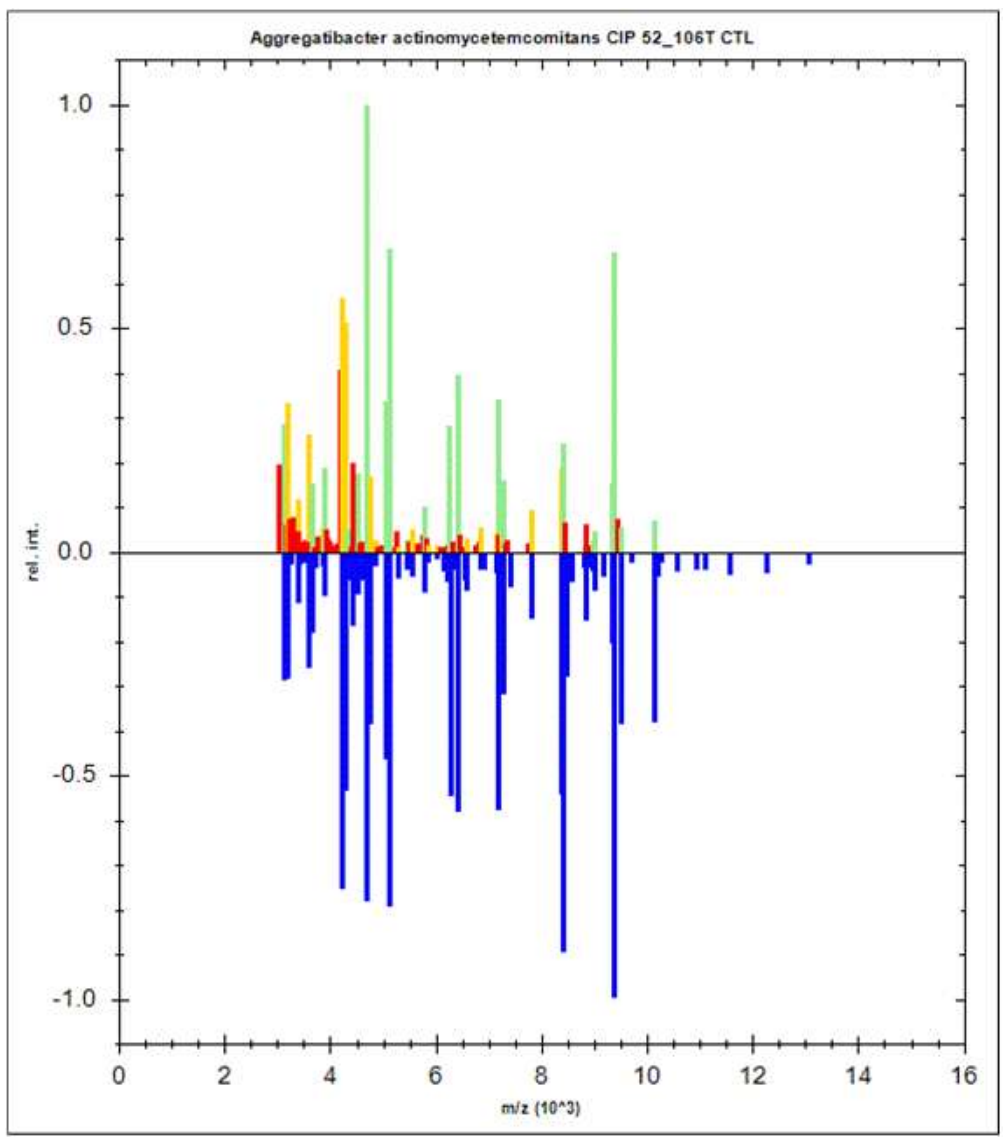

A. actinomycetemcomitans HK1651 (JP2 clone representative) *vs* Bruker MSP (*A. actinomycetemcomitans*) CIP 52_106T CTL

Log score value 2.15

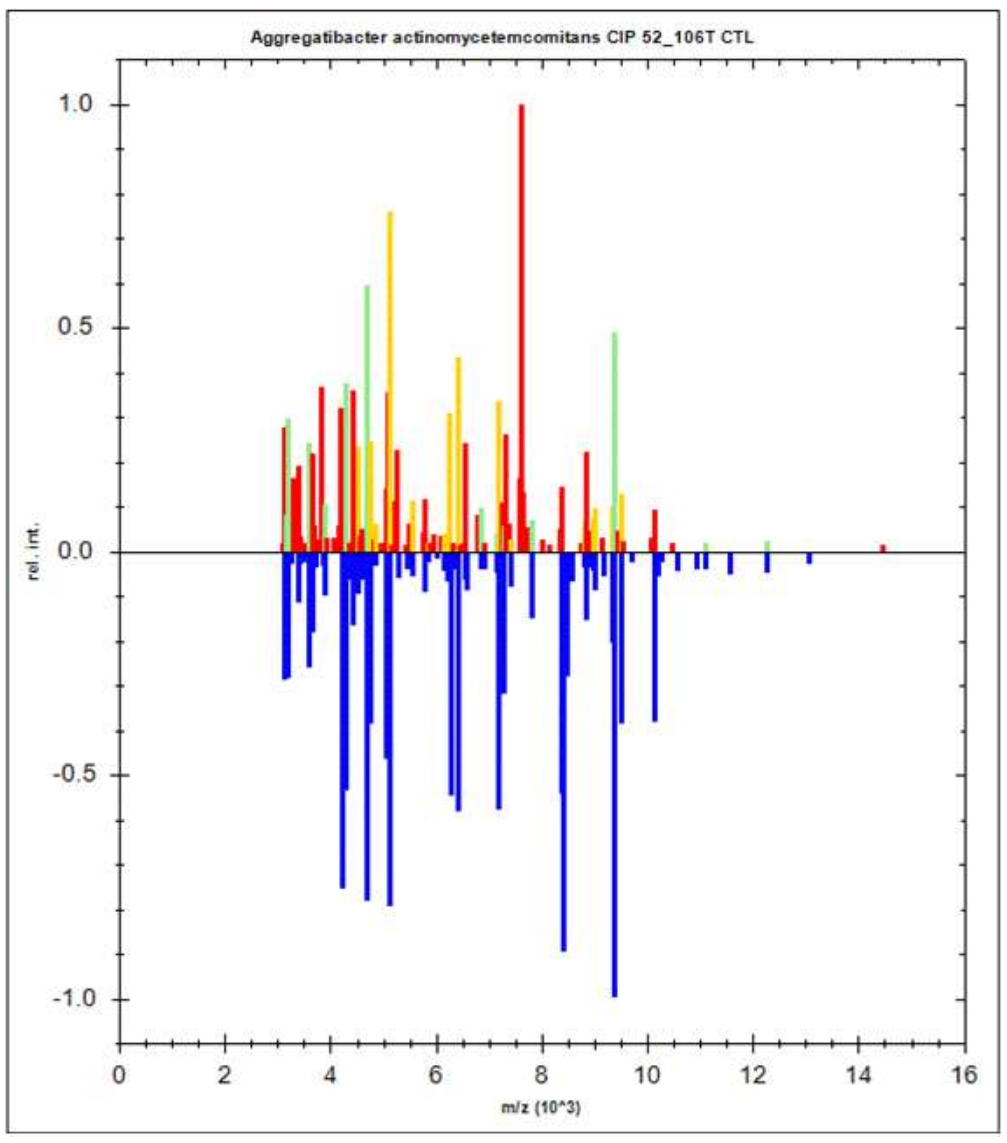

A. animalimorsus PN491 *vs* Bruker MSP (*A. actinomycetemcomitan*) CIP 52_106T CTL

Log score value 1.82

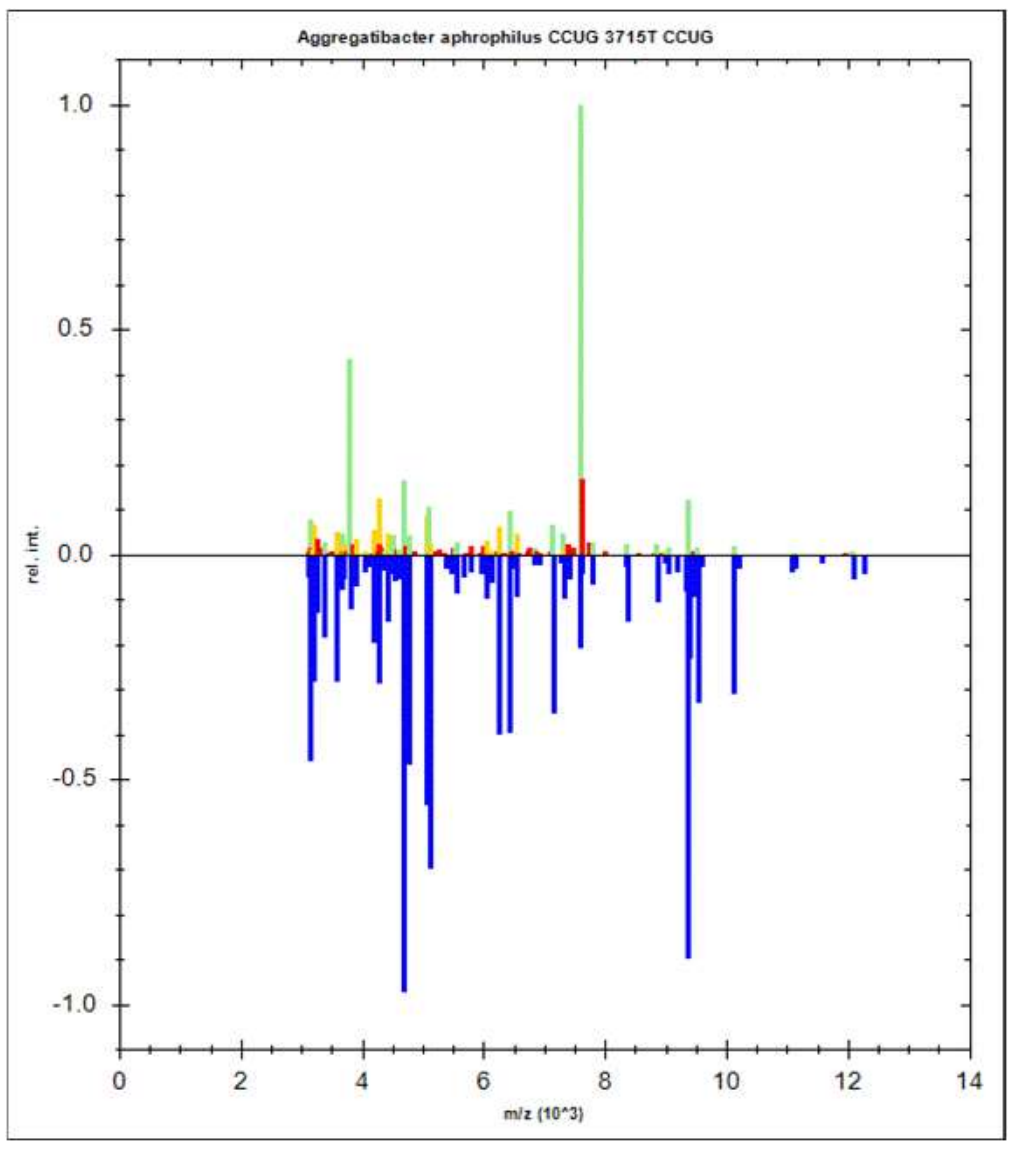

A. aphrophilus ATCC33389^{T} *vs* Bruker MSP (*A. aphrophilus*) CCUG 3715T CCUG

Log score value 2.12

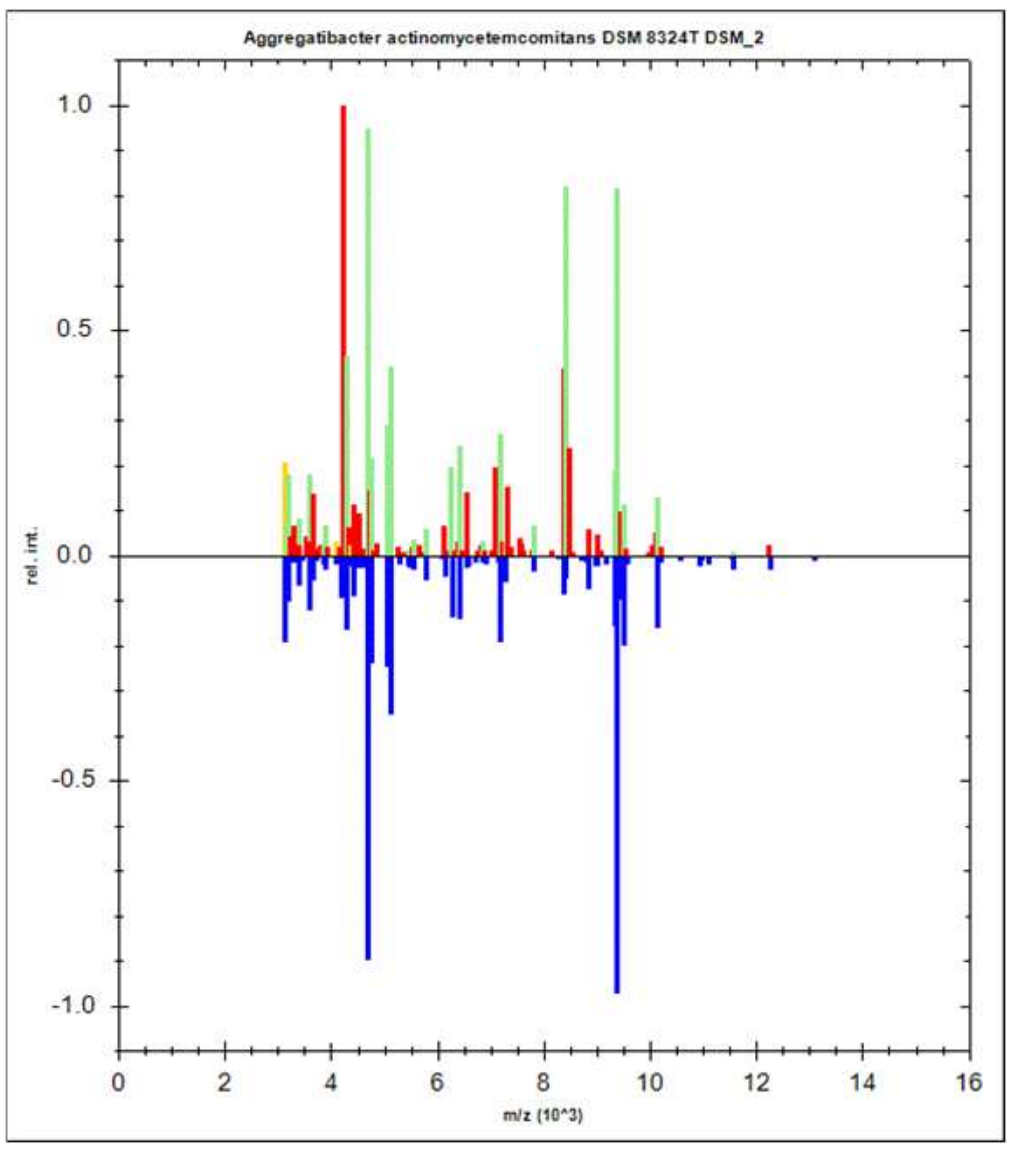

A. holmi PH791/56^{T} *vs* Bruker MSP (*A. actinomycetemcomitans*) DSM 8324T DSM_2

Log score value 1.97

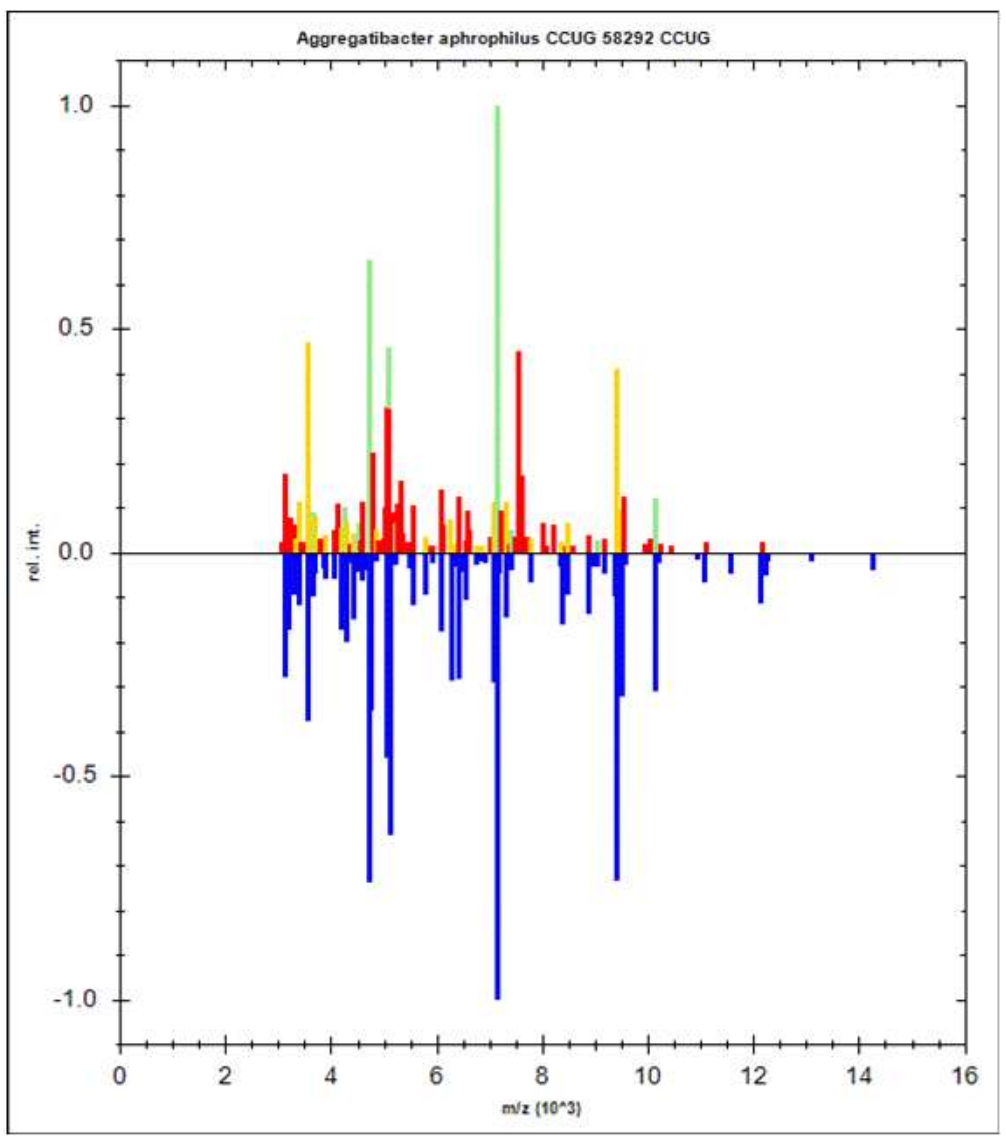

A. kilianii PN528^{T} *vs* Bruker MSP (*A. aphrophilus*) CCUG 58292 CCUG

Log score value 1.89

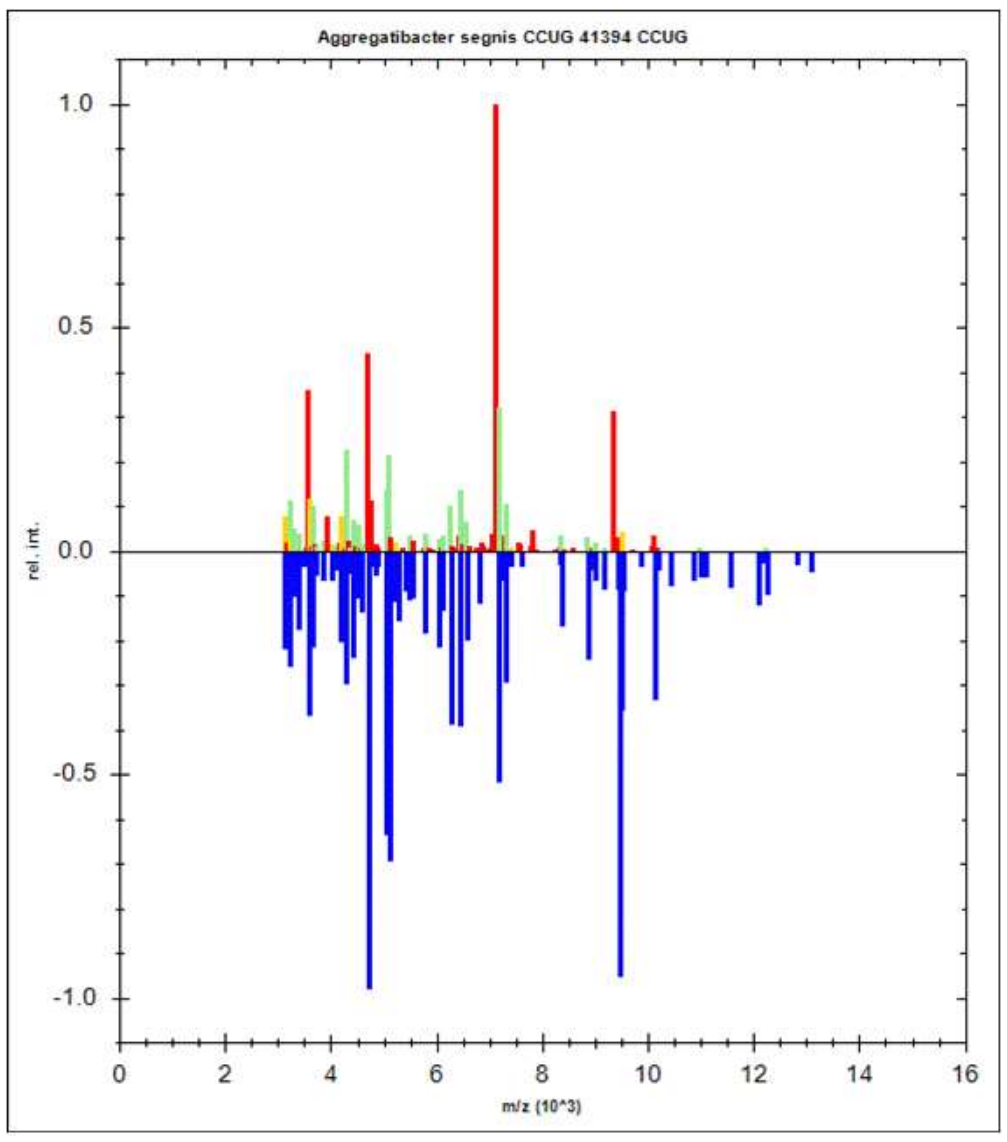

A. segnis ATCC 33393^T *vs* Bruker MSP (*A. segnis*) CCUG 41394 CCUG.

Log score value 2.16

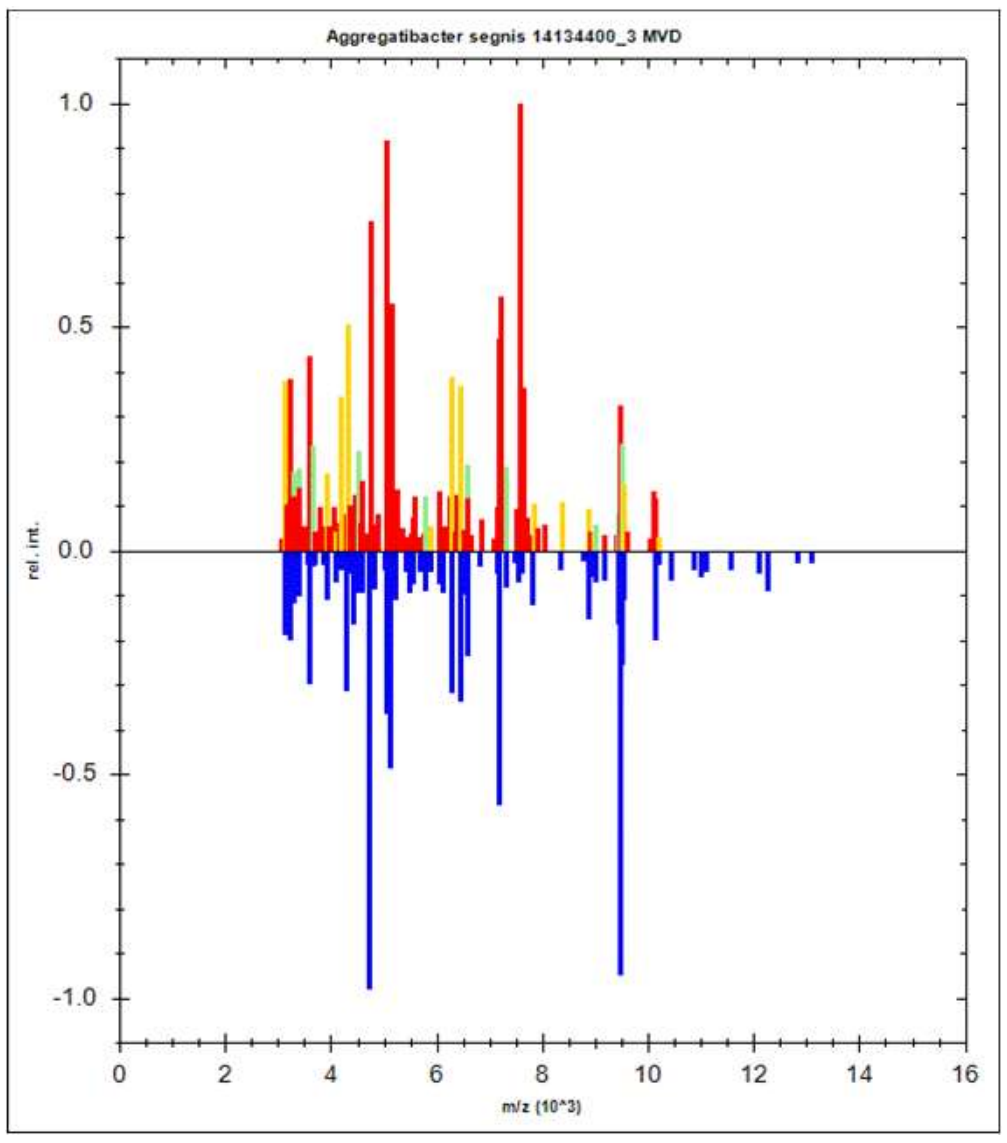

A. valvarum HerlE20^T *vs* Bruker MSP *(A. segnis)* 14134400_3 MVD

Log score value 1.59

Supplementary Figure 3. nadV sequence alignment.

MAFFT 7.526 (--auto --adjustdirection) alignment of 21 nadV homologs (18 full-length + 3 truncated), 1389 alignment columns.
Truncated A. aphrophilus forms (ATCC7901, CCUG11575, NCTC10557) appear as short sequences flanked by gaps. The 14 nadV-absent genomes are not shown.
Colours: A green, C blue, G gold, T red, gap white.

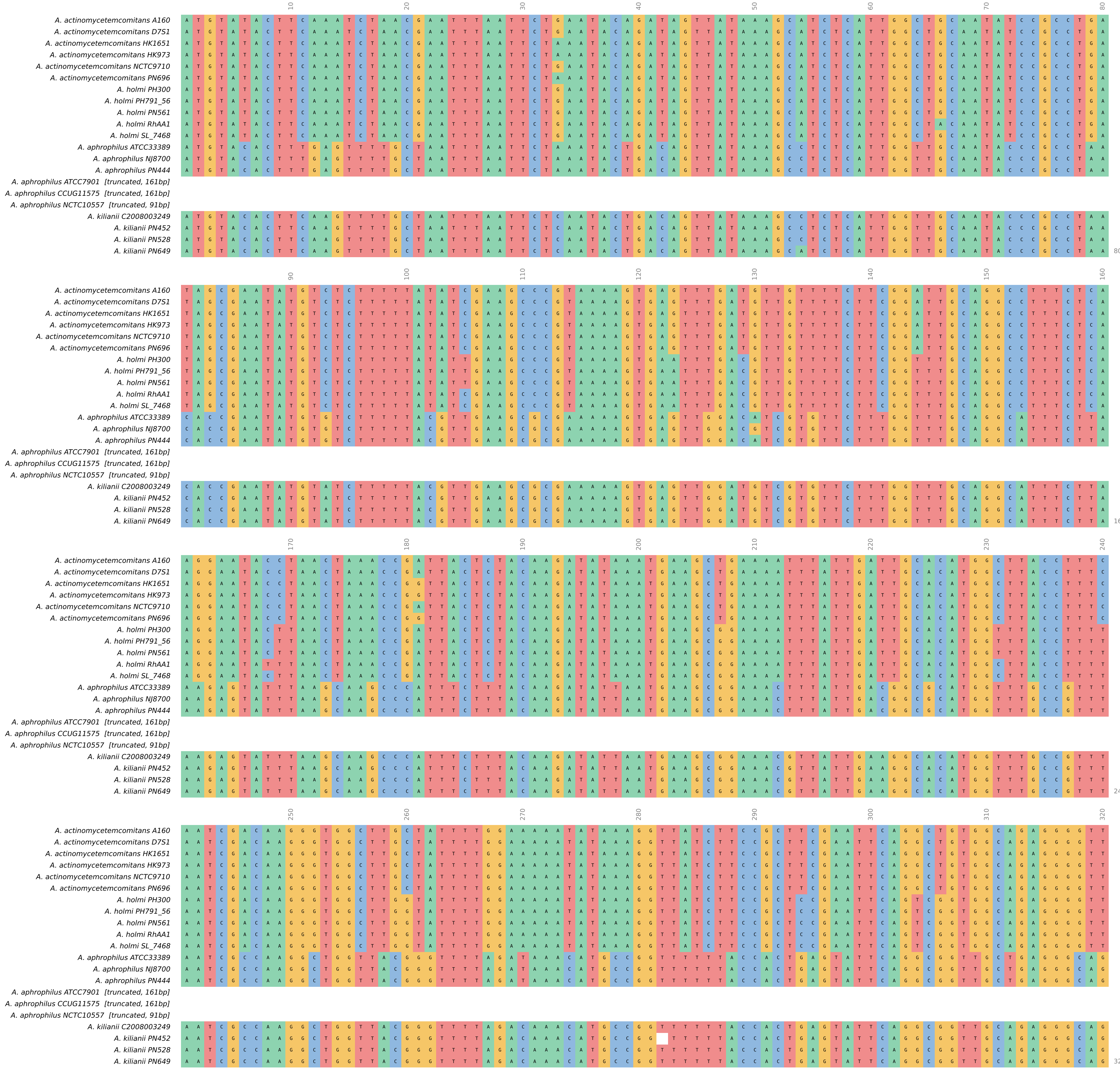

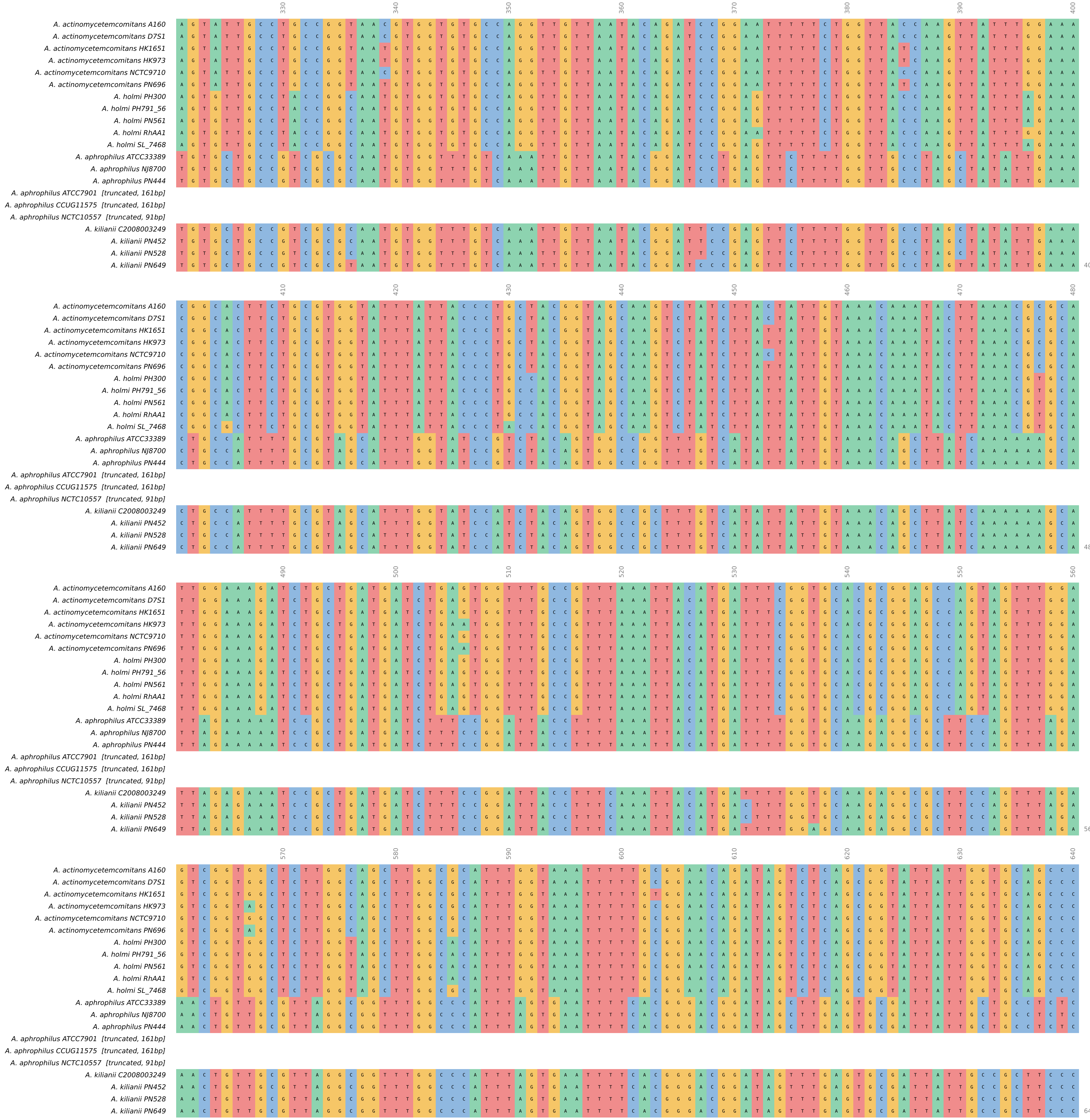
330
340
350
360
370
380
390
400
410
420
430
440
450
460
470
480
490
500
510
520
530
540
550
560
570
580
590
600
610
620
630
640
A. actinomycetemcomitans A160
A. actinomycetemcomitans D7S1
A. actinomycetemcomitans HK1651
A. actinomycetemcomitans HK973
A. actinomycetemcomitans NCTC9710
A. actinomycetemcomitans PN696
A. holmi PH300
A. holmi PH791_56
A. holmi PN561
A. holmi RhAA1
A. holmi SL_7468
A. aphrophilus ATCC33389
A. aphrophilus NJ8700
A. aphrophilus PN444
A. aphrophilus ATCC7901 [truncated, 161bp]
A. aphrophilus CCUG11575 [truncated, 161bp]
A. aphrophilus NCTC10557 [truncated, 91bp]
A. kilianii C2008003249
A. kilianii PN452
A. kilianii PN528
A. kilianii PN649

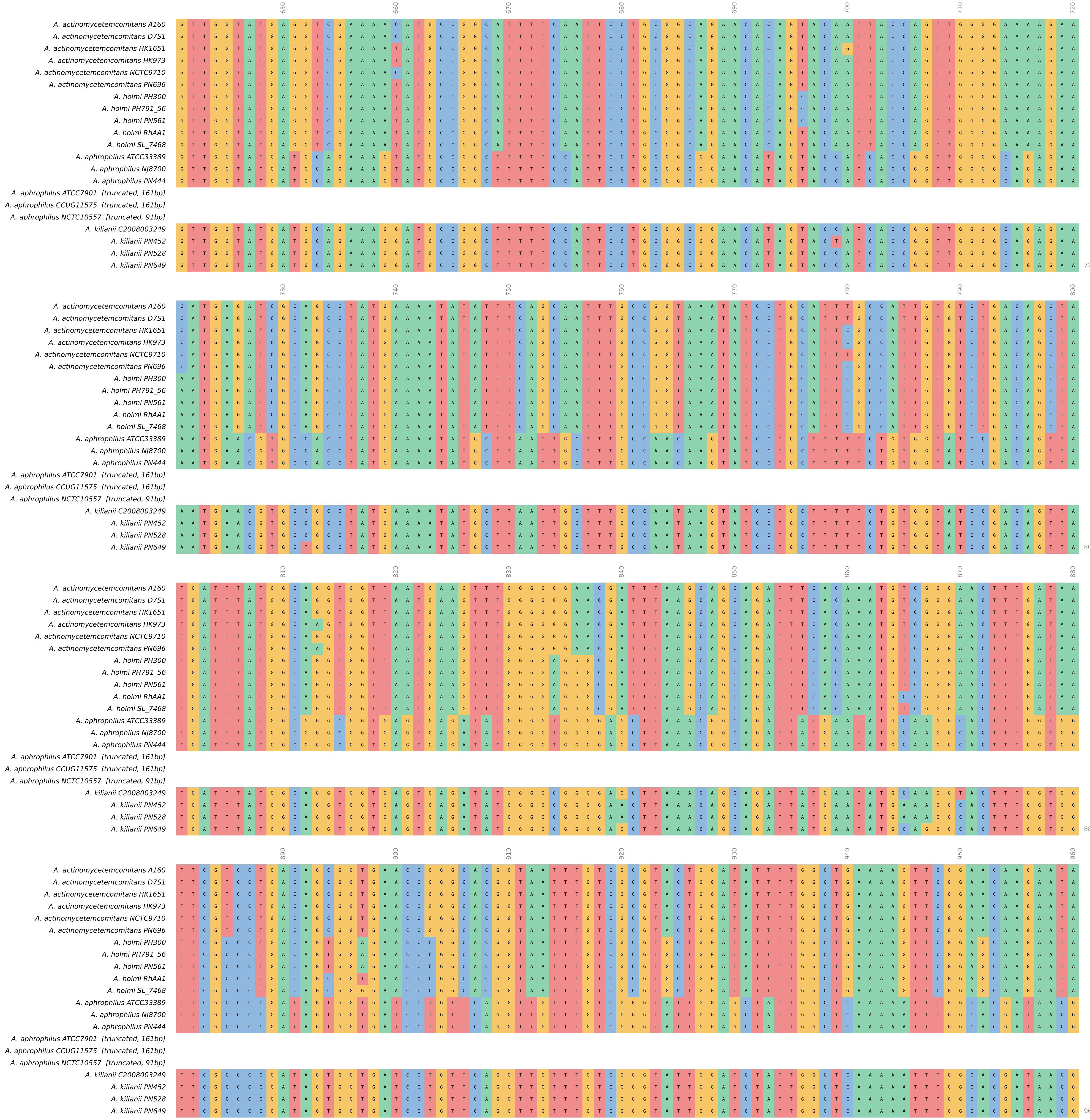

650
660
670
680
690
700
710
720
A. actinomycetemcomitans A160
A. actinomycetemcomitans D7S1
A. actinomycetemcomitans HK1651
A. actinomycetemcomitans HK973
A. actinomycetemcomitans NCTC9710
A. actinomycetemcomitans PN696
A. holmi PH300
A. holmi PH791_56
A. holmi PN561
A. holmi RhAA1
A. holmi SL_7468
A. aphrophilus ATCC33389
A. aphrophilus NJ8700
A. aphrophilus PN444
A. aphrophilus ATCC7901 [truncated, 161bp]
A. aphrophilus CCUG11575 [truncated, 161bp]
A. aphrophilus NCTC10557 [truncated, 91bp]
A. kilianii C2008003249
A. kilianii PN452
A. kilianii PN528
A. kilianii PN649
72
730
740
750
760
770
780
790
800
A. actinomycetemcomitans A160
A. actinomycetemcomitans D7S1
A. actinomycetemcomitans HK1651
A. actinomycetemcomitans HK973
A. actinomycetemcomitans NCTC9710
A. actinomycetemcomitans PN696
A. holmi PH300
A. holmi PH791_56
A. holmi PN561
A. holmi RhAA1
A. holmi SL_7468
A. aphrophilus ATCC33389
A. aphrophilus NJ8700
A. aphrophilus PN444
A. aphrophilus ATCC7901 [truncated, 161bp]
A. aphrophilus CCUG11575 [truncated, 161bp]
A. aphrophilus NCTC10557 [truncated, 91bp]
A. kilianii C2008003249
A. kilianii PN452
A. kilianii PN528
A. kilianii PN649
80
810
820
830
840
850
860
870
880
A. actinomycetemcomitans A160
A. actinomycetemcomitans D7S1
A. actinomycetemcomitans HK1651
A. actinomycetemcomitans HK973
A. actinomycetemcomitans NCTC9710
A. actinomycetemcomitans PN696
A. holmi PH300
A. holmi PH791_56
A. holmi PN561
A. holmi RhAA1
A. holmi SL_7468
A. aphrophilus ATCC33389
A. aphrophilus NJ8700
A. aphrophilus PN444
A. aphrophilus ATCC7901 [truncated, 161bp]
A. aphrophilus CCUG11575 [truncated, 161bp]
A. aphrophilus NCTC10557 [truncated, 91bp]
A. kilianii C2008003249
A. kilianii PN452
A. kilianii PN528
A. kilianii PN649
88
890
900
910
920
930
940
950
960
A. actinomycetemcomitans A160
A. actinomycetemcomitans D7S1
A. actinomycetemcomitans HK1651
A. actinomycetemcomitans HK973
A. actinomycetemcomitans NCTC9710
A. actinomycetemcomitans PN696
A. holmi PH300
A. holmi PH791_56
A. holmi PN561
A. holmi RhAA1
A. holmi SL_7468
A. aphrophilus ATCC33389
A. aphrophilus NJ8700
A. aphrophilus PN444
A. aphrophilus ATCC7901 [truncated, 161bp]
A. aphrophilus CCUG11575 [truncated, 161bp]
A. aphrophilus NCTC10557 [truncated, 91bp]
A. kilianii C2008003249
A. kilianii PN452
A. kilianii PN528
A. kilianii PN649
96

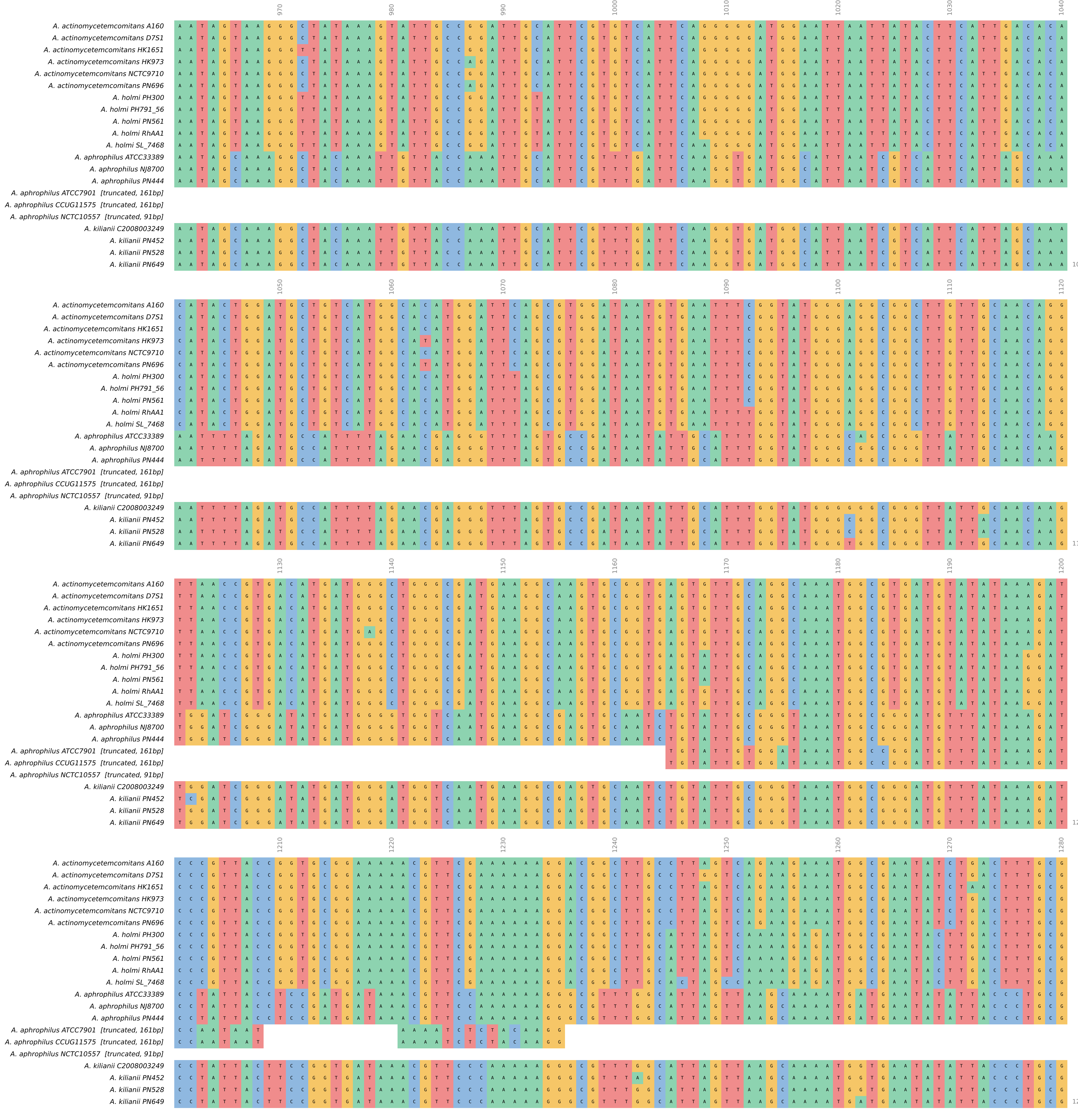
970
980
990
1000
1010
1020
1030
1040
A. actinomycetemcomitans A160
A. actinomycetemcomitans D7S1
A. actinomycetemcomitans HK1651
A. actinomycetemcomitans HK973
A. actinomycetemcomitans NCTC9710
A. actinomycetemcomitans PN696
A. holmi PH300
A. holmi PH791_56
A. holmi PN561
A. holmi RhAA1
A. holmi SL_7468
A. aphrophilus ATCC33389
A. aphrophilus NJ8700
A. aphrophilus PN444
A. aphrophilus ATCC7901 [truncated, 161bp]
A. aphrophilus CCUG11575 [truncated, 161bp]
A. aphrophilus NCTC10557 [truncated, 91bp]
A. kilianii C2008003249
A. kilianii PN452
A. kilianii PN528
A. kilianii PN649
1050
1060
1070
1080
1090
1100
1110
1120
1130
1140
1150
1160
1170
1180
1190
1200
1210
1220
1230
1240
1250
1260
1270
1280

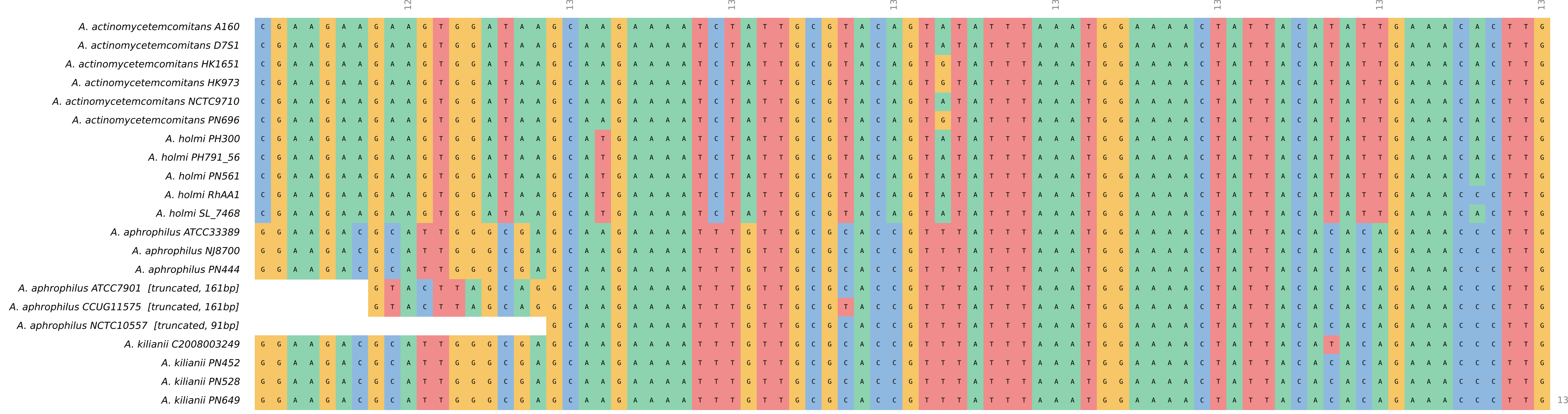

A. actinomycetemcomitans A160
A. actinomycetemcomitans D7S1
A. actinomycetemcomitans HK1651
A. actinomycetemcomitans HK973
A. actinomycetemcomitans NCTC9710
A. actinomycetemcomitans PN696
A. holmi PH300
A. holmi PH791_56
A. holmi PN561
A. holmi RhAA1
A. holmi SL_7468
A. aphrophilus ATCC33389
A. aphrophilus NJ8700
A. aphrophilus PN444
A. aphrophilus ATCC7901 [truncated, 161bp]
A. aphrophilus CCUG11575 [truncated, 161bp]
A. aphrophilus NCTC10557 [truncated, 91bp]
A. kilianii C2008003249
A. kilianii PN452
A. kilianii PN528
A. kilianii PN649

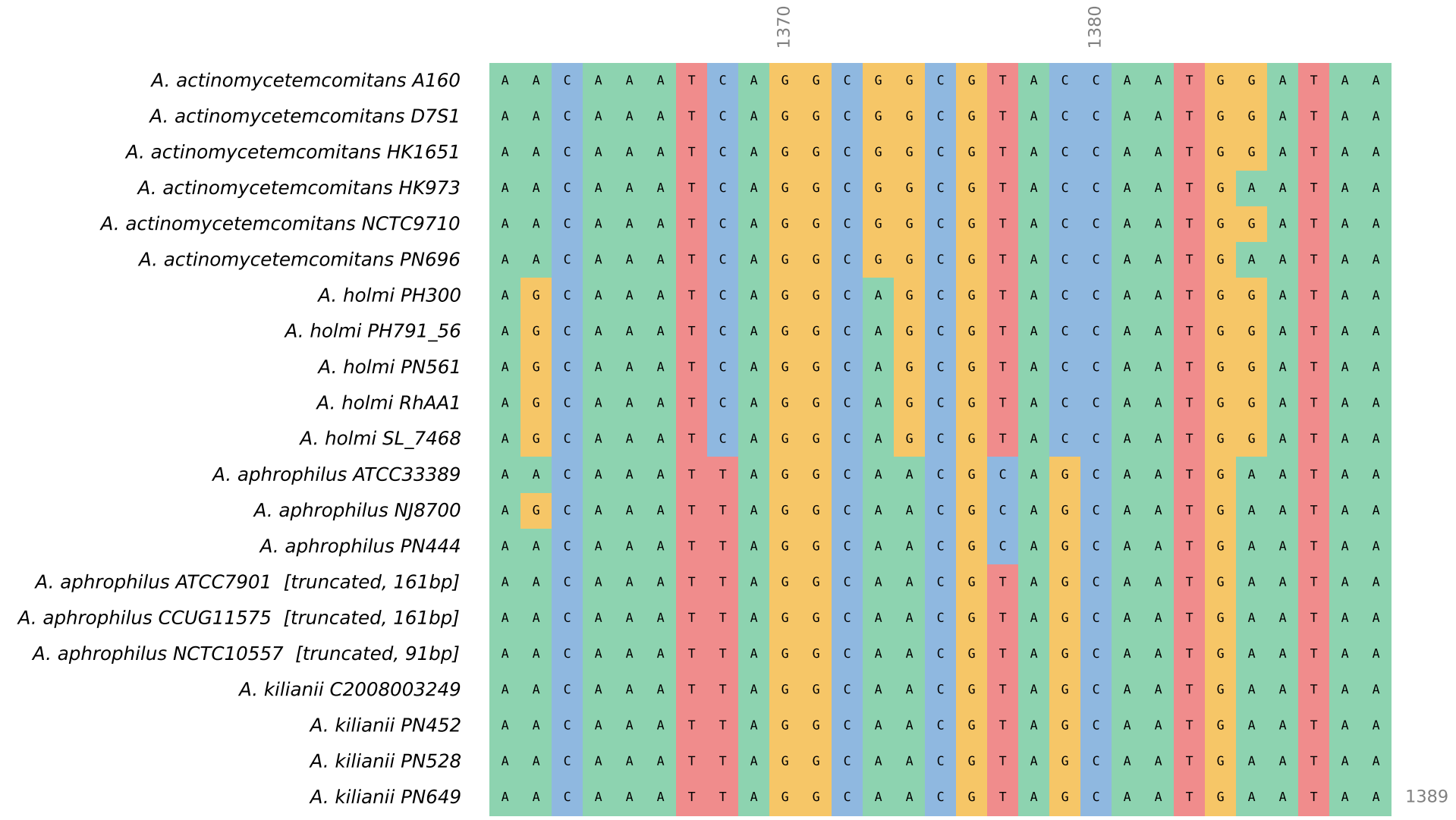

1370
1380
A. actinomycetemcomitans A160
A. actinomycetemcomitans D7S1
A. actinomycetemcomitans HK1651
A. actinomycetemcomitans HK973
A. actinomycetemcomitans NCTC9710
A. actinomycetemcomitans PN696
A. holmi PH300
A. holmi PH791_56
A. holmi PN561
A. holmi RhAA1
A. holmi SL_7468
A. aphrophilus ATCC33389
A. aphrophilus NJ8700
A. aphrophilus PN444
A. aphrophilus ATCC7901 [truncated, 161bp]
A. aphrophilus CCUG11575 [truncated, 161bp]
A. aphrophilus NCTC10557 [truncated, 91bp]
A. kilianii C2008003249
A. kilianii PN452
A. kilianii PN528
A. kilianii PN649
1389

Supplemental Figure S4. Colony appearance on chocolate agar (8.2 cm plate)

20 h

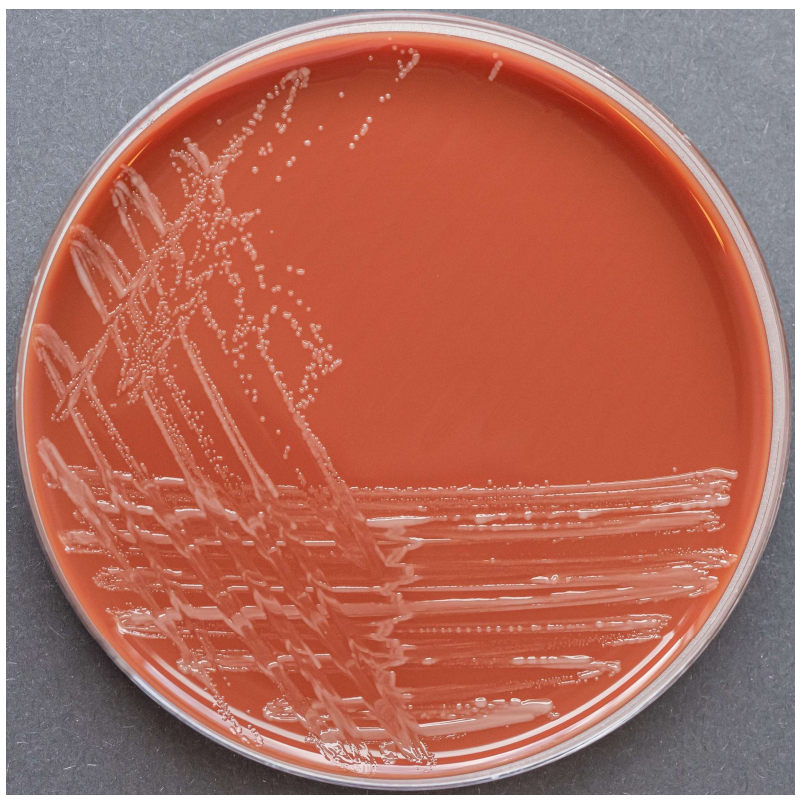

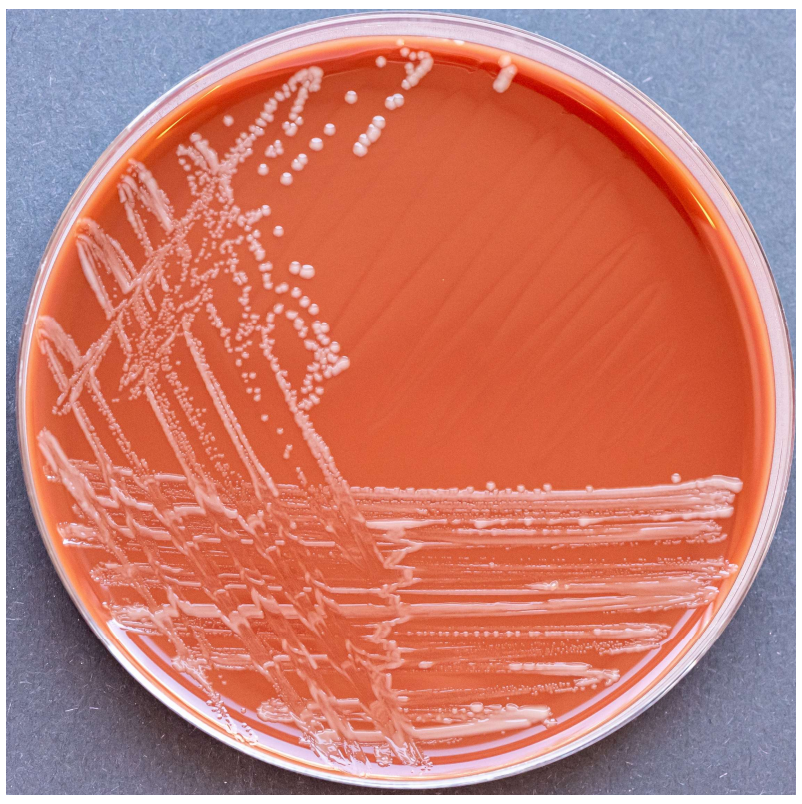

44 h

A. actinomycetemcomitans NCTC9710[T]; type strain of type species

20 h

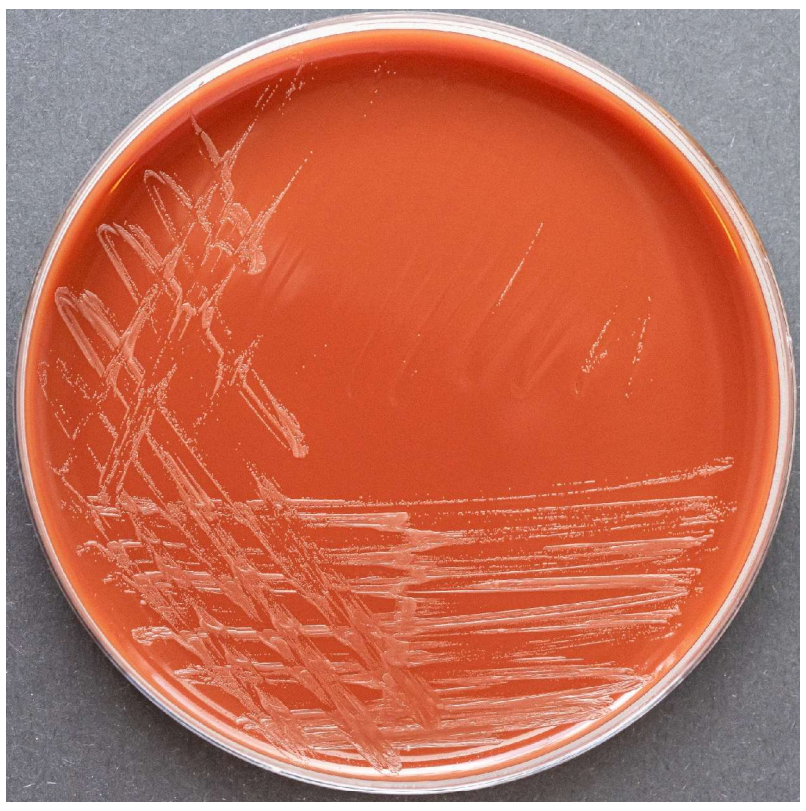

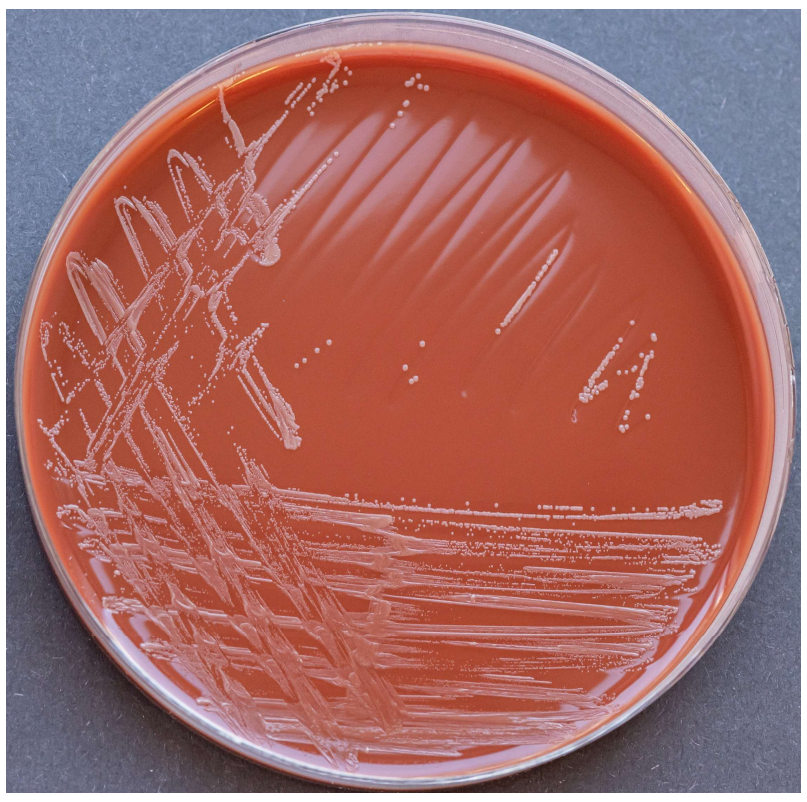

44 h

A. actinomycetemcomitans HK1651; representative of the JP2 clone

20 h

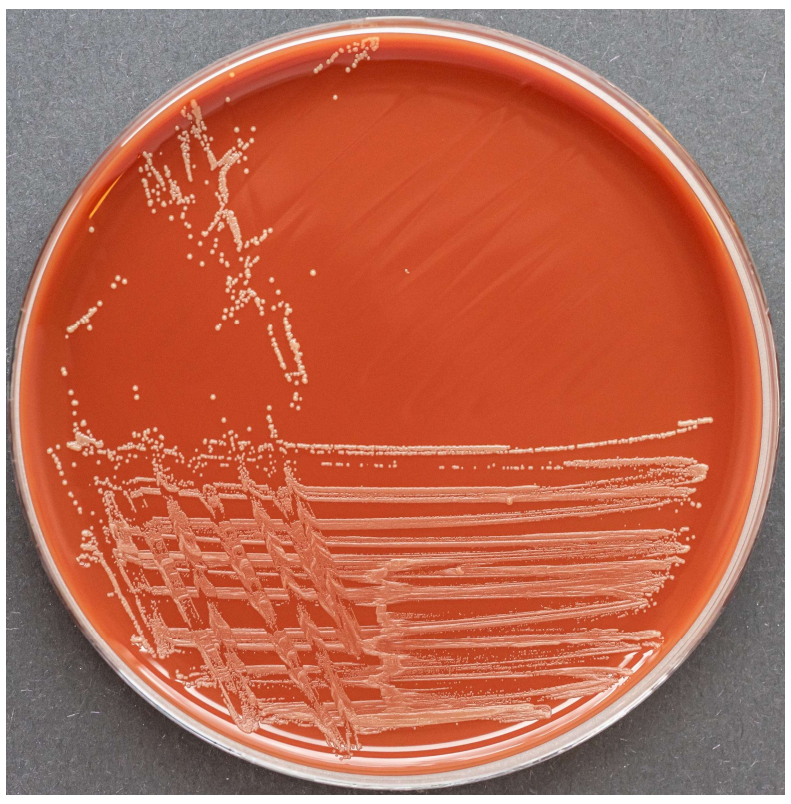

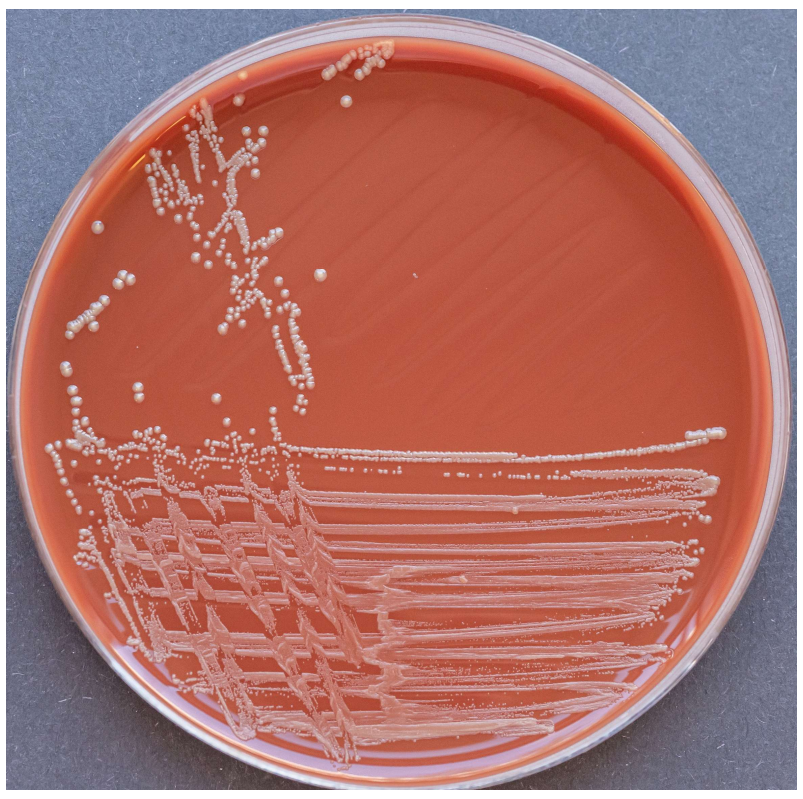

44 h

A. animalimorsus PN491[T]; type strain of species

20 h

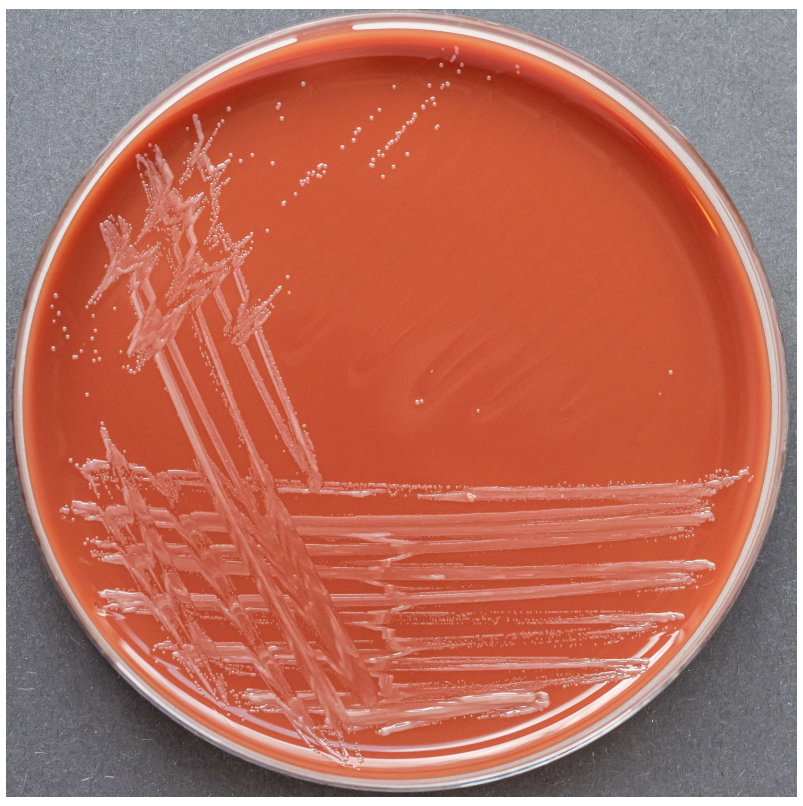

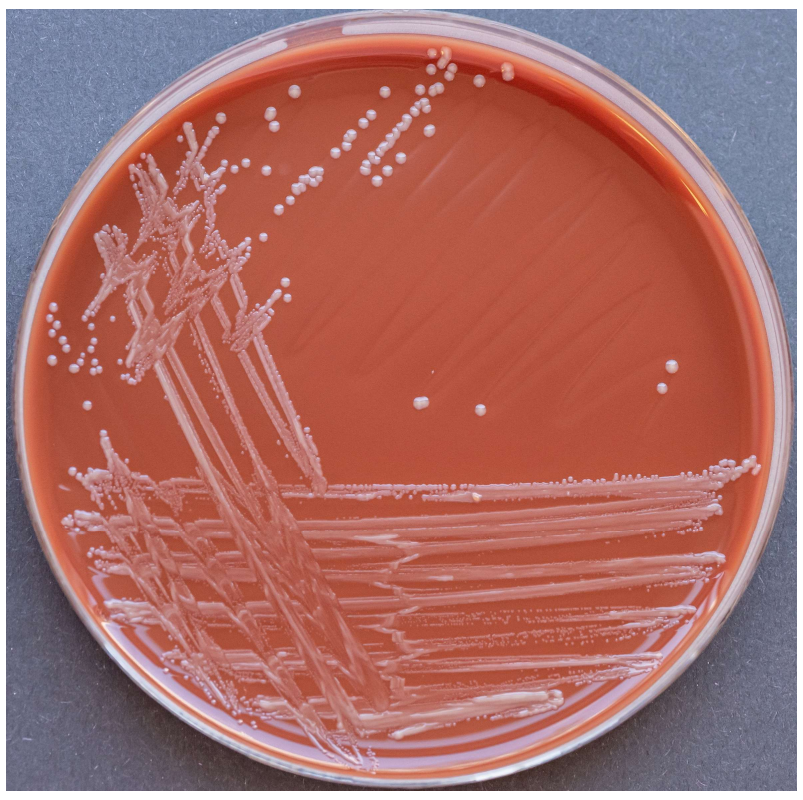

44 h

A. aphrophilus ATCC33389[T]; type strain of species

20 h

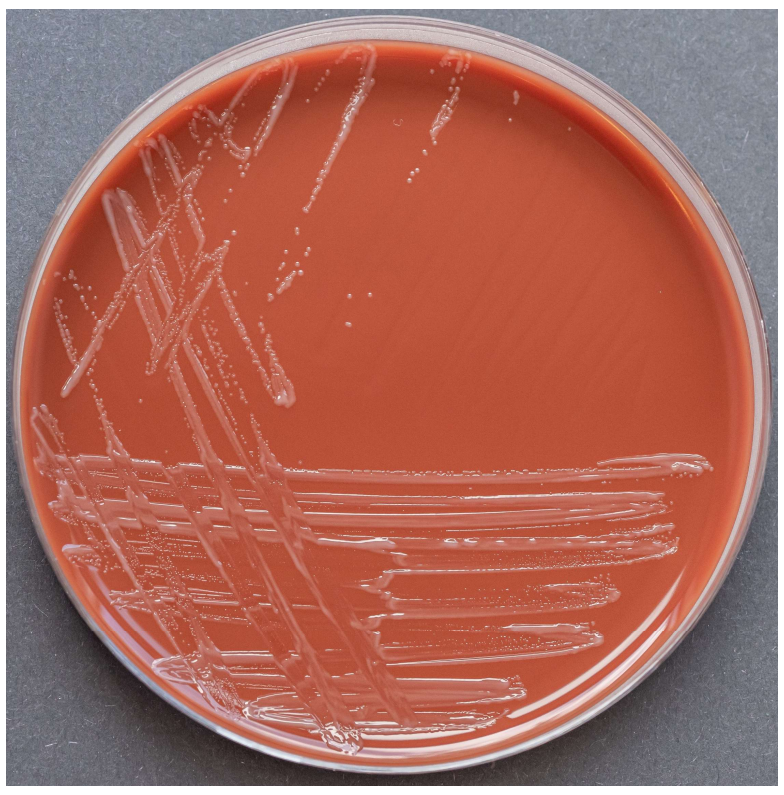

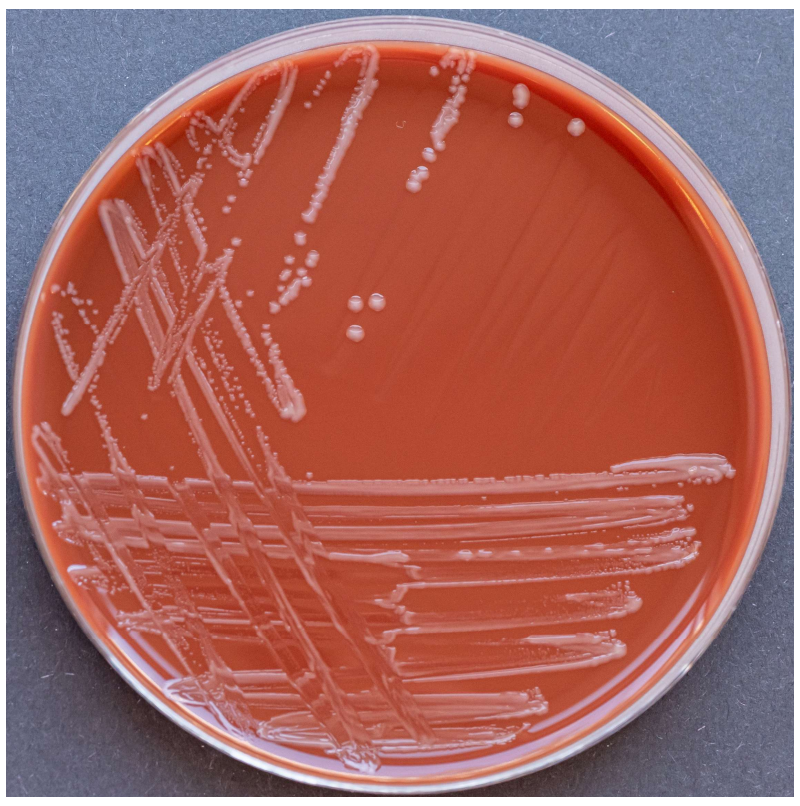

44 h

A. holmi PH791/56^{T}; type strain of species

20 h

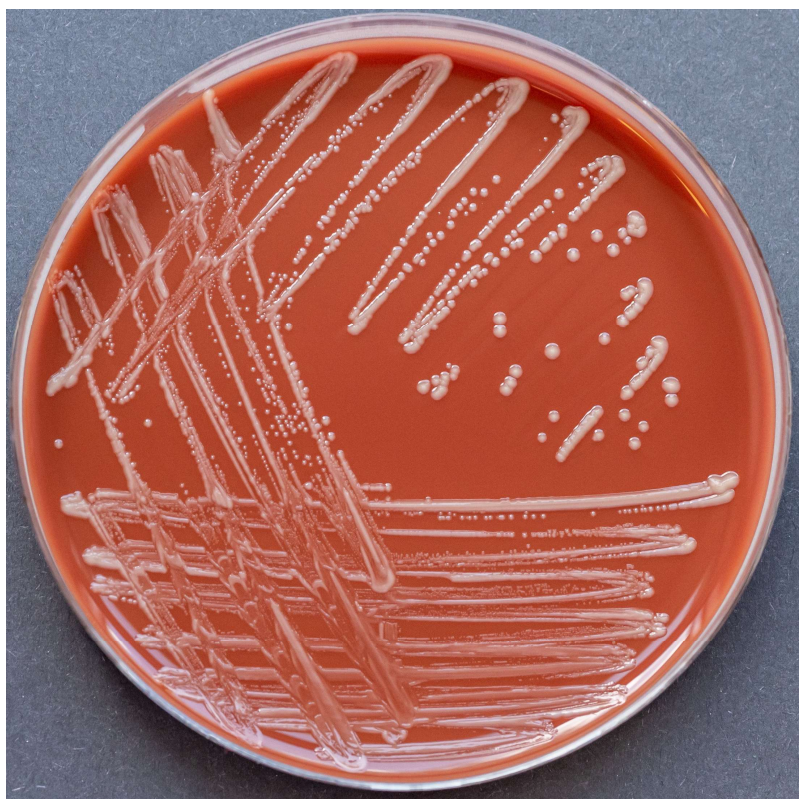

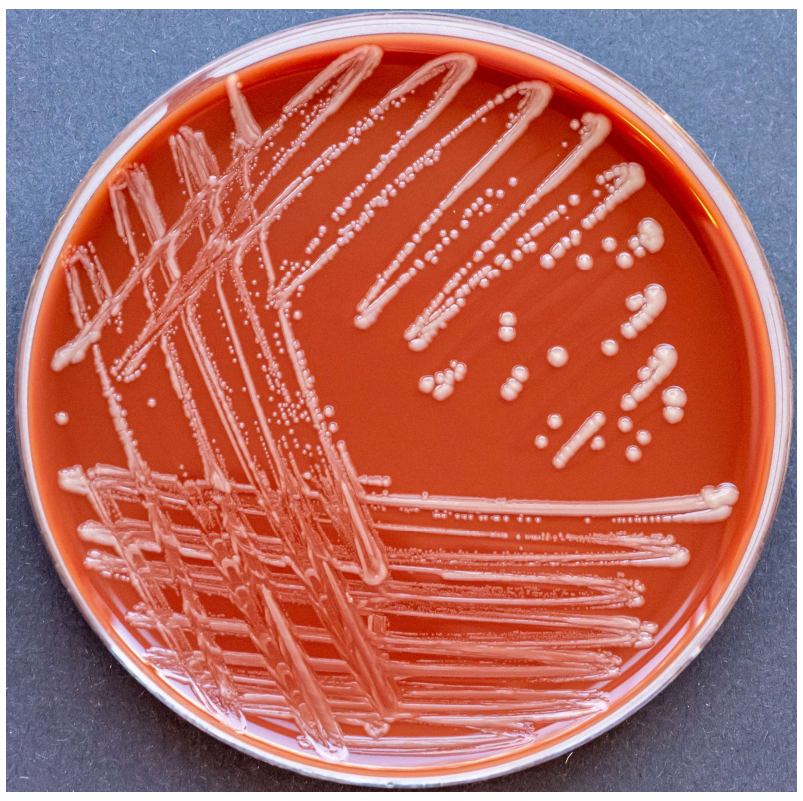

44 h

A. kilianii PN528^{T}; type strain of species

20 h

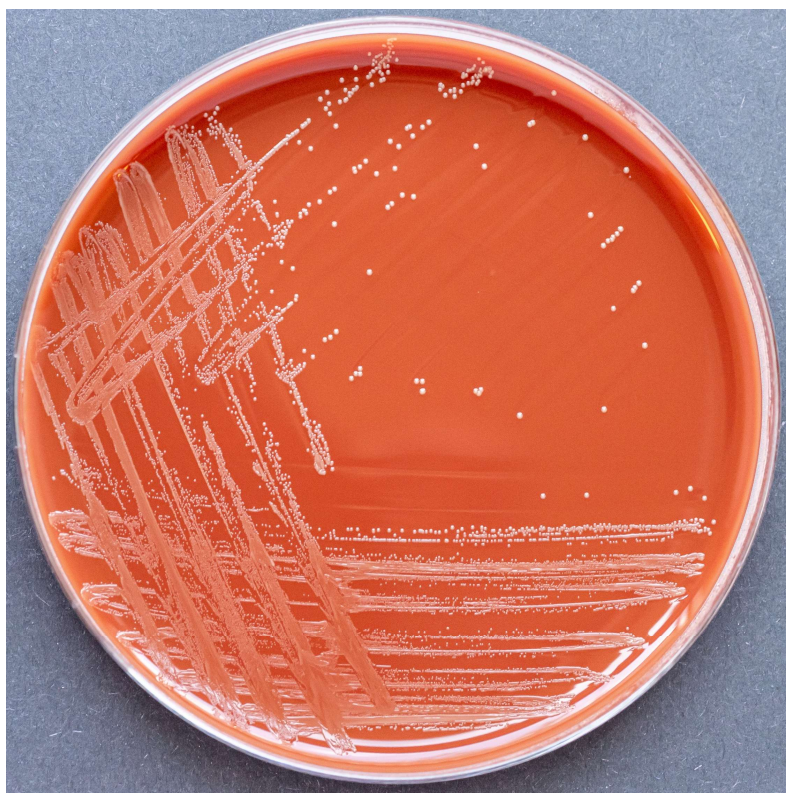

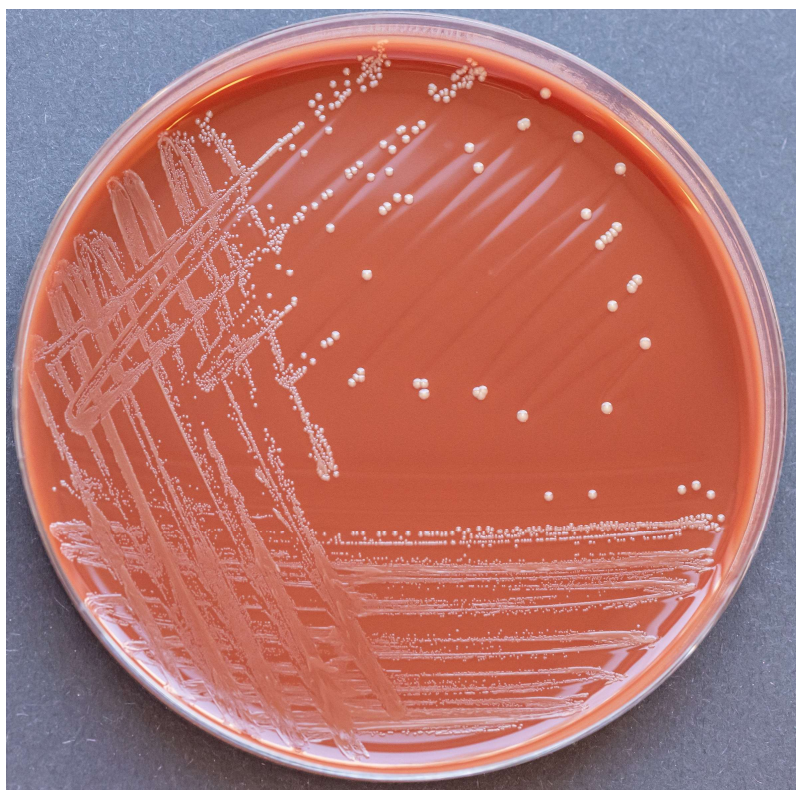

44 h

A. segnis ATCC 33393^{T}; type strain of species

20 h

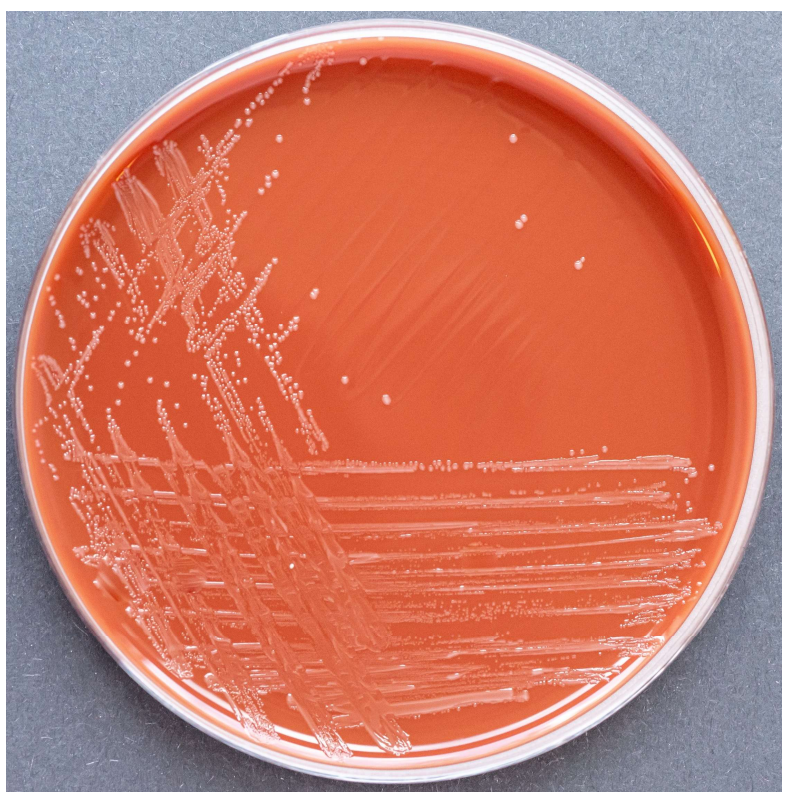

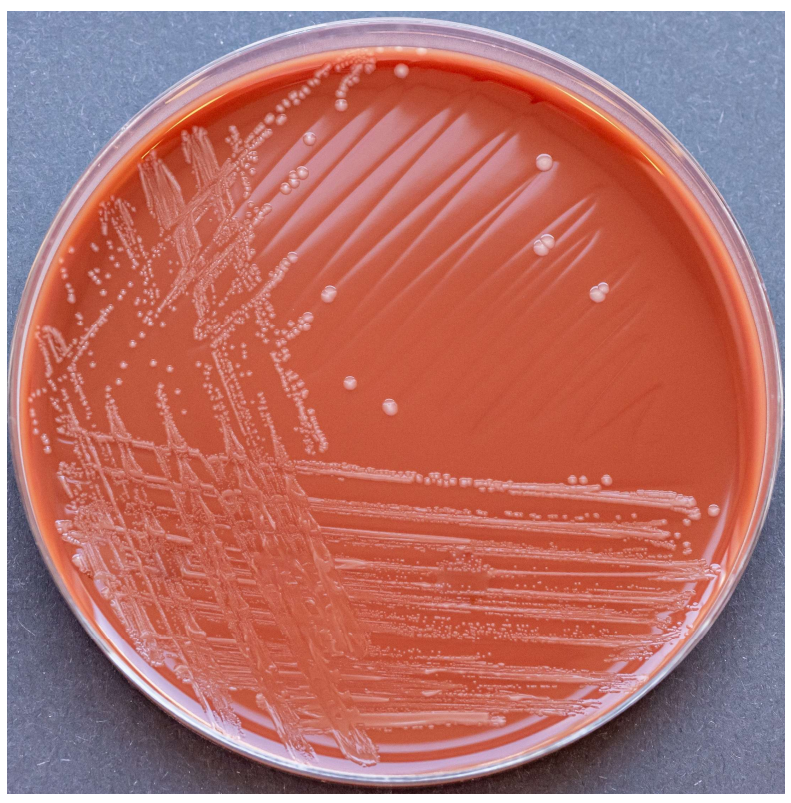

44 h

A. valvarum HerlE20^{T}; type strain of species
