# Supplementary material for: Distribution of nicotinamide phosphoribosyltransferase in genus Aggregatibacter and description of Aggregatibacter animalimorsus sp. nov., Aggregatibacter holmi sp. nov., and Aggregatibacter valvarum sp. nov
Source: Front Microbiol. 2026 Jul 29;17:1866808. doi: 10.3389/fmicb.2026.1866808 (PMC13461592; doi:10.3389/fmicb.2026.1866808)
Supplement: Supplementary file 2 [file Data_Sheet_2.PDF]

```
# packages in environment at /home/kyg9yd/miniforge3/envs/aggre_r226:
#
# Name                                       Version          Build
 Channel
_libgcc_mutex                                0.1              conda_forge
 conda-forge
_openmp_mutex                                4.5              2_gnu
 conda-forge
alsa-lib                                     1.2.11           hd590300_1
 conda-forge
aragorn                                      1.2.41           h7b50bb2_5
 bioconda
argtable2                                    2.13             hd590300_1004
 conda-forge
barrnap                                      0.9              hdfd78af_4
 bioconda
bedtools                                     2.31.1           hf5e1c6e_2
 bioconda
blast                                        2.16.0           hc155240_3
 bioconda
blast-legacy                                 2.2.26           h9ee0642_4
 bioconda
bzip2                                        1.0.8            hda65f42_8
 conda-forge
c-ares                                       1.34.6           hb03c661_0
 conda-forge
ca-certificates                              2025.11.12       hbd8a1cb_0
 conda-forge
cairo                                        1.18.0           h3faef2a_0
 conda-forge
clustalo                                     1.2.4            h503566f_10
 bioconda
clustalw                                     2.1              h9948957_12
 bioconda
consan                                       1.2              h7b50bb2_7
 bioconda
curl                                         8.8.0            he654da7_1
 conda-forge
dendropy                                     5.0.8            pyhdfd78af_1
 bioconda
dialign-tx                                   1.0.2            h7b50bb2_8
 bioconda
dnspython                                    2.8.0            pyhcf101f3_0
 conda-forge
entrez-direct                                22.4             he881be0_0
 bioconda
expat                                        2.7.3            hecca717_0
 conda-forge
famsa                                        2.2.3            h9948957_1
 bioconda
fasttree                                     2.2.0            h7b50bb2_1
 bioconda
font-ttf-dejavu-sans-mono                    2.37             hab24e00_0
 conda-forge
```

```
font-ttf-inconsolata                    3.000                h77eed37_0
 conda-forge
font-ttf-source-code-pro                2.038                h77eed37_0
 conda-forge
font-ttf-ubuntu                         0.83                 h77eed37_3
 conda-forge
fontconfig                              2.14.2               h14ed4e7_0
 conda-forge
fonts-conda-ecosystem                   1                    0
 conda-forge
fonts-conda-forge                       1                    hc364b38_1
 conda-forge
freetype                                2.12.1               h267a509_2
 conda-forge
gawk                                    5.3.1                hcd3d067_0
 conda-forge
giflib                                  5.2.2                hd590300_0
 conda-forge
gmp                                     6.3.0                hac33072_2
 conda-forge
graphite2                               1.3.14               hecca717_2
 conda-forge
gsl                                     2.7                  he838d99_0
 conda-forge
harfbuzz                                9.0.0                hfac3d4d_0
 conda-forge
hmmer                                   3.4                  hb6cb901_4
 bioconda
icu                                     73.2                 h59595ed_0
 conda-forge
infernal                                1.1.5                pl5321h7b50bb2_4
 bioconda
kalign2                                 2.04                 h7b50bb2_8
 bioconda
keyutils                                1.6.3                hb9d3cd8_0
 conda-forge
krb5                                    1.21.3               h659f571_0
 conda-forge
lcms2                                   2.16                 hb7c19ff_0
 conda-forge
ld_impl_linux-64                        2.45                 bootstrap_ha15bf96_4
 conda-forge
lerc                                    4.0.0                h0aef613_1
 conda-forge
libasprintf                             0.25.1               h3f43e3d_1
 conda-forge
libblas                                 3.11.0               4_h4a7cf45_openblas
 conda-forge
libcblas                                3.11.0               4_h0358290_openblas
 conda-forge
libcups                                 2.3.3                h4637d8d_4
 conda-forge
libcurl                                 8.8.0                hca28451_1
 conda-forge
```

```
libdb                                     6.2.32           h9c3ff4c_0
 conda-forge
libdeflate                                1.20             hd590300_0
 conda-forge
libedit                                   3.1.20250104     pl5321h7949ede_0
 conda-forge
libev                                     4.33             hd590300_2
 conda-forge
libexpat                                  2.7.3            hecca717_0
 conda-forge
libffi                                    3.5.2            h9ec8514_0
 conda-forge
libgcc                                    15.2.0           he0feb66_16
 conda-forge
libgcc-ng                                 15.2.0           h69a702a_16
 conda-forge
libgettextpo                              0.25.1           h3f43e3d_1
 conda-forge
libgfortran                               15.2.0           h69a702a_16
 conda-forge
libgfortran-ng                            15.2.0           h69a702a_16
 conda-forge
libgfortran5                              15.2.0           h68bc16d_16
 conda-forge
libglib                                   2.80.2           hf974151_0
 conda-forge
libgomp                                   15.2.0           he0feb66_16
 conda-forge
libiconv                                  1.18             h3b78370_2
 conda-forge
libidn11                                  1.34             h1cef754_0
 conda-forge
libidn2                                   2.3.8            hfac485b_1
 conda-forge
libjpeg-turbo                             3.1.2            hb03c661_0
 conda-forge
liblzma                                   5.8.1            hb9d3cd8_2
 conda-forge
liblzma-devel                             5.8.1            hb9d3cd8_2
 conda-forge
libnghttp2                                1.58.0           h47da74e_1
 conda-forge
libnsl                                    2.0.1            hb9d3cd8_1
 conda-forge
libopenblas                               0.3.30           pthreads_h94d23a6_4
 conda-forge
libpng                                    1.6.43           h2797004_0
 conda-forge
libsqlite                                 3.46.0           hde9e2c9_0
 conda-forge
libssh2                                   1.11.0           h0841786_0
 conda-forge
libstdcxx                                 15.2.0           h934c35e_16
 conda-forge
```

```
libstdcxx-ng                                15.2.0               hdf11a46_16
 conda-forge
libtiff                                     4.6.0                h1dd3fc0_3
 conda-forge
libunistring                                0.9.10               h7f98852_0
 conda-forge
libuuid                                     2.41.2               h5347b49_1
 conda-forge
libwebp-base                                1.6.0                hd42ef1d_0
 conda-forge
libxcb                                      1.15                 h0b41bf4_0
 conda-forge
libxcrypt                                   4.4.36               hd590300_1
 conda-forge
libzlib                                     1.2.13               h4ab18f5_6
 conda-forge
mafft                                       7.526                h4bc722e_0
 conda-forge
minced                                      0.4.2                hdfd78af_1
 bioconda
mpfr                                        4.2.1                h90cbb55_3
 conda-forge
mpi                                         1.0                  openmpi
 conda-forge
muscle                                      3.8.1551             h9948957_9
 bioconda
mustang                                     3.2.4                h9948957_0
 bioconda
ncbi-vdb                                    3.3.0                h9948957_0
 bioconda
ncurses                                     6.5                  h2d0b736_3
 conda-forge
openjdk                                     22.0.1               hb622114_0
 conda-forge
openmpi                                     4.1.6                hc5af2df_101
 conda-forge
openssl                                     3.6.0                h26f9b46_0
 conda-forge
paml                                        4.10.9               h7b50bb2_1
 bioconda
parallel                                    20251122             ha770c72_0
 conda-forge
pasta                                       1.9.2                py312hccd54bf_5
 bioconda
pcre                                        8.45                 h9c3ff4c_0
 conda-forge
pcre2                                       10.43                hcad00b1_0
 conda-forge
perl                                        5.32.1               7_hd590300_perl5
 conda-forge
perl-algorithm-diff                         1.201                pl5321hd8ed1ab_0
 conda-forge
perl-archive-tar                            3.04                 pl5321hdfd78af_0
 bioconda
```

```
perl-b-cow                                0.007             pl5321hb9d3cd8_1
 conda-forge
perl-base                                 2.23              pl5321hd8ed1ab_0
 conda-forge
perl-bio-asn1-entrezgene                  1.73              pl5321hdfd78af_3
 bioconda
perl-bio-coordinate                       1.007001          pl5321hdfd78af_3
 bioconda
perl-bio-featureio                        1.6.905           pl5321hdfd78af_4
 bioconda
perl-bio-samtools                         1.43              pl5321he4a0461_4
 bioconda
perl-bio-searchio-hmmer                   1.7.3             pl5321hdfd78af_0
 bioconda
perl-bio-tools-phylo-paml                 1.7.3             pl5321hdfd78af_3
 bioconda
perl-bio-tools-run-alignment-clustalw     1.7.4             pl5321hdfd78af_3
 bioconda
perl-bio-tools-run-alignment-tcoffee      1.7.4             pl5321hdfd78af_5
 bioconda
perl-bioperl                              1.7.8             hdfd78af_1
 bioconda
perl-bioperl-core                         1.7.8             pl5321hdfd78af_1
 bioconda
perl-bioperl-run                          1.007003          pl5321hdfd78af_0
 bioconda
perl-business-isbn                        3.007             pl5321hd8ed1ab_0
 conda-forge
perl-business-isbn-data                   20210112.006      pl5321hd8ed1ab_0
 conda-forge
perl-carp                                 1.50              pl5321hd8ed1ab_0
 conda-forge
perl-class-data-inheritable               0.09              pl5321ha770c72_0
 conda-forge
perl-clone                                0.46              pl5321hb9d3cd8_1
 conda-forge
perl-common-sense                         3.75              pl5321hd8ed1ab_0
 conda-forge
perl-compress-raw-bzip2                   2.214             pl5321hda65f42_0
 conda-forge
perl-compress-raw-zlib                    2.214             pl5321h4dac143_0
 conda-forge
perl-constant                             1.33              pl5321hd8ed1ab_0
 conda-forge
perl-data-dump                            1.25              pl5321h7b50bb2_2
 bioconda
perl-data-dumper                          2.183             pl5321hb9d3cd8_1
 conda-forge
perl-db_file                              1.858             pl5321hb9d3cd8_0
 conda-forge
perl-devel-stacktrace                     2.04              pl5321h296ab09_0
 conda-forge
perl-digest-hmac                          1.05              pl5321hdfd78af_0
 bioconda
```

perl-digest-md5	2.59	pl5321hb9d3cd8_3	conda-forge
perl-encode	3.21	pl5321hb9d3cd8_1	conda-forge
perl-encode-locale	1.05	pl5321hdfd78af_7	bioconda
perl-exception-class	1.45	pl5321ha770c72_0	conda-forge
perl-exporter	5.74	pl5321hd8ed1ab_0	conda-forge
perl-exporter-tiny	1.002002	pl5321hd8ed1ab_0	conda-forge
perl-extutils-makemaker	7.70	pl5321hd8ed1ab_0	conda-forge
perl-file-listing	6.16	pl5321hdfd78af_0	bioconda
perl-file-path	2.18	pl5321hd8ed1ab_0	conda-forge
perl-file-slurper	0.014	pl5321hdfd78af_0	bioconda
perl-file-sort	1.01	pl5321hdfd78af_3	bioconda
perl-file-spec	3.48_01	pl5321hdfd78af_2	bioconda
perl-file-temp	0.2304	pl5321hd8ed1ab_0	conda-forge
perl-getopt-long	2.58	pl5321hdfd78af_0	bioconda
perl-html-parser	3.81	pl5321h4ac6f70_1	bioconda
perl-html-tagset	3.24	pl5321hdfd78af_0	bioconda
perl-http-cookiejar-lwp	0.014	pl5321hdfd78af_0	bioconda
perl-http-cookies	6.11	pl5321hdfd78af_0	bioconda
perl-http-daemon	6.16	pl5321hdfd78af_0	bioconda
perl-http-date	6.06	pl5321hdfd78af_0	bioconda
perl-http-message	7.01	pl5321hdfd78af_0	bioconda
perl-http-negotiate	6.01	pl5321hdfd78af_4	bioconda
perl-inc-latest	0.500	pl5321ha770c72_0	conda-forge
perl-io-compress	2.214	pl5321h503566f_0	bioconda
perl-io-html	1.004	pl5321hdfd78af_0	bioconda
perl-io-socket-ssl	2.075	pl5321hd8ed1ab_0	conda-forge
perl-io-string	1.08	pl5321hdfd78af_4	bioconda

perl-io-tty	1.20	pl5321hb9d3cd8_3	conda-forge
perl-io-zlib	1.15	pl5321hdfd78af_1	bioconda
perl-ipc-run	20250809.0	pl5321hdfd78af_0	bioconda
perl-json	4.10	pl5321hdfd78af_1	bioconda
perl-json-xs	4.04	pl5321h9948957_0	bioconda
perl-libwww-perl	6.81	pl5321hdfd78af_0	bioconda
perl-libxml-perl	0.08	pl5321hdfd78af_3	bioconda
perl-list-moreutils	0.430	pl5321hdfd78af_0	bioconda
perl-list-moreutils-xs	0.430	pl5321h7b50bb2_5	bioconda
perl-lwp-mediatypes	6.04	pl5321hdfd78af_1	bioconda
perl-lwp-protocol-https	6.14	pl5321hdfd78af_1	bioconda
perl-mime-base64	3.16	pl5321hb9d3cd8_3	conda-forge
perl-module-build	0.4234	pl5321ha770c72_1	conda-forge
perl-module-load	0.34	pl5321hdfd78af_0	bioconda
perl-mozilla-ca	20250602	pl5321hdfd78af_0	bioconda
perl-net-http	6.24	pl5321hdfd78af_0	bioconda
perl-net-ssleay	1.92	pl5321hf14f497_1	conda-forge
perl-ntlm	1.09	pl5321hdfd78af_5	bioconda
perl-parent	0.243	pl5321hd8ed1ab_0	conda-forge
perl-pathtools	3.75	pl5321hb9d3cd8_2	conda-forge
perl-scalar-list-utils	1.70	pl5321hb03c661_0	conda-forge
perl-socket	2.027	pl5321h5c03b87_6	bioconda
perl-storable	3.15	pl5321hb9d3cd8_2	conda-forge
perl-sub-uplevel	0.2800	pl5321hb9d3cd8_0	conda-forge
perl-test-deep	1.130	pl5321hd8ed1ab_0	conda-forge
perl-test-differences	0.72	pl5321ha770c72_0	conda-forge
perl-test-exception	0.43	pl5321hd8ed1ab_0	conda-forge

perl-test-fatal	0.016	pl5321hdfd78af_0	bioconda
perl-test-most	0.38	pl5321hdfd78af_0	bioconda
perl-test-needs	0.002009	pl5321hd8ed1ab_0	conda-forge
perl-test-requiresinternet	0.05	pl5321hdfd78af_1	bioconda
perl-test-warn	0.37	pl5321hd8ed1ab_0	conda-forge
perl-test-warnings	0.031	pl5321ha770c72_0	conda-forge
perl-text-diff	1.45	pl5321hd8ed1ab_0	conda-forge
perl-time-local	1.35	pl5321hdfd78af_0	bioconda
perl-timedate	2.33	pl5321hdfd78af_2	bioconda
perl-tree-dag_node	1.35	pl5321hdfd78af_0	bioconda
perl-try-tiny	0.32	pl5321ha770c72_1	conda-forge
perl-types-serialiser	1.01	pl5321hdfd78af_0	bioconda
perl-uri	5.34	pl5321ha770c72_0	conda-forge
perl-url-encode	0.03	pl5321h9ee0642_1	bioconda
perl-www-robotrules	6.02	pl5321hdfd78af_4	bioconda
perl-xml-dom	1.46	pl5321hdfd78af_1	bioconda
perl-xml-dom-xpath	0.14	pl5321hdfd78af_2	bioconda
perl-xml-namespacesupport	1.12	pl5321hd8ed1ab_0	conda-forge
perl-xml-parser	2.44_01	pl5321hf2e2c51_1004	conda-forge
perl-xml-regexp	0.04	pl5321hdfd78af_3	bioconda
perl-xml-sax	1.02	pl5321hd8ed1ab_0	conda-forge
perl-xml-sax-base	1.09	pl5321hd8ed1ab_0	conda-forge
perl-xml-sax-expat	0.51	pl5321hd8ed1ab_0	conda-forge
perl-xml-simple	2.25	pl5321hdfd78af_2	bioconda
perl-xml-xpathengine	0.14	pl5321hdfd78af_3	bioconda
phylip	3.697	h7b50bb2_3	bioconda
pip	25.3	pyh8b19718_0	conda-forge

```
pixman                                   0.46.4            h54a6638_1
 conda-forge
poa                                      2.0               h7b50bb2_6
 bioconda
prank                                    170427            h9948957_1
 bioconda
probcons                                 1.12              h8b12597_1
 bioconda
probconsrna                              1.10              h9f5acd7_4
 bioconda
proda                                    1.0               h503566f_7
 bioconda
prodigal                                 2.6.3             h7b50bb2_10
 bioconda
prokka                                   1.14.6            pl5321hdfd78af_5
 bioconda
pthread-stubs                            0.4               hb9d3cd8_1002
 conda-forge
pymongo                                  4.15.5            py312h1289d80_0
 conda-forge
python                                   3.12.3            hab00c5b_0_cpython
 conda-forge
python_abi                               3.12              8_cp312
 conda-forge
raxml                                    8.2.13            h7b50bb2_3
 bioconda
readline                                 8.2               h8c095d6_2
 conda-forge
rpsbproc                                 0.5.0             h6a68c12_1
 bioconda
sap                                      1.1.3             h7b50bb2_5
 bioconda
setuptools                               80.9.0            pyhff2d567_0
 conda-forge
sniffio                                  1.3.1             pyhd8ed1ab_2
 conda-forge
t-coffee                                 13.46.2.7c9e712d  pl5321hb2a3317_0
 bioconda
tbl2asn-forever                          25.7.2f           h7b50bb2_6
 bioconda
tk                                       8.6.13            noxft_h4845f30_101
 conda-forge
tmalign                                  20170708          h8d36177_7
 bioconda
tzdata                                   2025b             h78e105d_0
 conda-forge
viennarna                                2.7.0             py312pl5321h9f0f9fd_0
 bioconda
wget                                     1.21.4            hda4d442_0
 conda-forge
wheel                                    0.45.1            pyhd8ed1ab_1
 conda-forge
xorg-fixesproto                          5.0               hb9d3cd8_1003
 conda-forge
```

```
xorg-inputproto                          2.3.2               hb9d3cd8_1003
 conda-forge
xorg-kbproto                             1.0.7               hb9d3cd8_1003
 conda-forge
xorg-libice                              1.1.2               hb9d3cd8_0
 conda-forge
xorg-libsm                               1.2.6               he73a12e_0
 conda-forge
xorg-libx11                              1.8.9               h8ee46fc_0
 conda-forge
xorg-libxau                              1.0.12              hb03c661_1
 conda-forge
xorg-libxdmcp                            1.1.5               hb03c661_1
 conda-forge
xorg-libxext                             1.3.4               h0b41bf4_2
 conda-forge
xorg-libxfixes                           5.0.3               h7f98852_1004
 conda-forge
xorg-libxi                               1.7.10              h4bc722e_1
 conda-forge
xorg-libxrender                          0.9.11              hd590300_0
 conda-forge
xorg-libxt                               1.3.0               hd590300_1
 conda-forge
xorg-libxtst                             1.2.5               h4bc722e_0
 conda-forge
xorg-recordproto                         1.14.2              hb9d3cd8_1003
 conda-forge
xorg-renderproto                         0.11.1              hb9d3cd8_1003
 conda-forge
xorg-xextproto                           7.3.0               hb9d3cd8_1004
 conda-forge
xorg-xproto                              7.0.31              hb9d3cd8_1008
 conda-forge
xz                                       5.8.1               hbcc6ac9_2
 conda-forge
xz-gpl-tools                             5.8.1               hbcc6ac9_2
 conda-forge
xz-tools                                 5.8.1               hb9d3cd8_2
 conda-forge
zlib                                     1.2.13              h4ab18f5_6
 conda-forge
zstd                                     1.5.6               ha6fb4c9_0
 conda-forge
```

```
# packages in environment at /home/kyg9yd/miniforge3/envs/aggre_phylo:
#
# Name                    Version          Build
Channel
_openmp_mutex             4.5              20_gnu
conda-forge
_r-mutex                  1.0.1            anacondar_1
conda-forge
alsa-lib                  1.2.16           hb03c661_0
conda-forge
any2fasta                 0.8.1            hdfd78af_0
bioconda
aragorn                   1.2.41           h7b50bb2_5
bioconda
archspec                  0.2.5            pyhd8ed1ab_0
conda-forge
argcomplete               3.6.3            pyhd8ed1ab_0
conda-forge
argh                      0.31.3           pyhd8ed1ab_1
conda-forge
argtable2                 2.13             hd590300_1004
conda-forge
aria2                     1.37.0           h4b33fff_4
conda-forge
arpack                    3.9.1            nompi_hf03ea27_102
conda-forge
backports.zstd            1.5.0            py311h6b1f9c4_0
conda-forge
barrnap                   1.10.6           pl5321hdfd78af_0
bioconda
bedtools                  2.31.1           h13024bc_3
bioconda
binutils_impl_linux-64    2.45.1           default_hfdba357_102
conda-forge
binutils_linux-64         2.45.1           default_h4852527_102
conda-forge
biocode                   0.12.1           pyhdfd78af_0
bioconda
bioconductor-ggtree       4.0.4            r45hdfd78af_1
bioconda
bioconductor-treeio       1.34.0           r45hdfd78af_0
bioconda
biopython                 1.87             py311h49ec1c0_0
conda-forge
blast                     2.17.0           h66d330f_0
bioconda
blast-legacy              2.2.26           hf7ff83a_5
bioconda
brotli                    1.2.0            hed03a55_1
conda-forge
brotli-bin                1.2.0            hb03c661_1
conda-forge
brotli-python             1.2.0            py311h66f275b_1
conda-forge
```

```
bwidget                                   1.10.1            ha770c72_1
conda-forge
bzip2                                     1.0.8             hda65f42_9
conda-forge
c-ares                                    1.34.6            hb03c661_0
conda-forge
ca-certificates                           2026.5.20         hbd8a1cb_0
conda-forge
cairo                                     1.18.4            he90730b_1
conda-forge
capnproto                                 1.0.2             h766bdaa_3
conda-forge
cd-hit                                    4.8.1             h5ca1c30_13
bioconda
certifi                                   2026.5.20         pyhd8ed1ab_0
conda-forge
charset-normalizer                        3.4.7             pyhd8ed1ab_0
conda-forge
clustalo                                  1.2.4             h503566f_10
bioconda
clustalw                                  2.1               h9948957_12
bioconda
contourpy                                 1.3.3             py311h724c32c_4
conda-forge
curl                                      8.20.0            hcf29cc6_0
conda-forge
cycler                                    0.12.1            pyhcf101f3_2
conda-forge
dendropy                                  5.0.8             pyhdfd78af_1
bioconda
diamond                                   2.2.1             he361c42_0
bioconda
entrez-direct                             24.0              he881be0_0
bioconda
expat                                     2.8.1             hecca717_0
conda-forge
fastani                                   1.34              hb66fcc3_6
bioconda
font-ttf-dejavu-sans-mono                 2.37              hab24e00_0
conda-forge
font-ttf-inconsolata                      3.000             h77eed37_0
conda-forge
font-ttf-source-code-pro                  2.038             h77eed37_0
conda-forge
font-ttf-ubuntu                           0.83              h77eed37_3
conda-forge
fontconfig                                2.18.1            h27c8c51_0
conda-forge
fonts-conda-ecosystem                     1                 0
conda-forge
fonts-conda-forge                         1                 hc364b38_1
conda-forge
fonttools                                 4.63.0            py311h3778330_0
conda-forge
```

freetype	2.14.3	ha770c72_0	conda-forge
fribidi	1.0.16	hb03c661_0	conda-forge
gawk	5.4.0	h0a3468a_0	conda-forge
gcc_impl_linux-64	15.2.0	he0086c7_19	conda-forge
gcc_linux-64	15.2.0	h7be306e_26	conda-forge
gffutils	0.14	pyh106432d_0	bioconda
gfortran_impl_linux-64	15.2.0	h281d09f_19	conda-forge
giflib	5.2.2	hd590300_0	conda-forge
glpk	5.0	h445213a_0	conda-forge
gmp	6.3.0	hac33072_2	conda-forge
gnutls	3.8.13	h18acefa_0	conda-forge
graphite2	1.3.15	hecca717_0	conda-forge
gsl	2.7	he838d99_0	conda-forge
gxx_impl_linux-64	15.2.0	hda75c37_19	conda-forge
gxx_linux-64	15.2.0	h92113df_26	conda-forge
gzip	1.14	hb9d3cd8_0	conda-forge
h2	4.3.0	pyhcf101f3_0	conda-forge
harfbuzz	14.2.1	h6083320_0	conda-forge
hmmer	3.4	h503566f_3	bioconda
hpack	4.1.0	pyhd8ed1ab_0	conda-forge
hyperframe	6.1.0	pyhd8ed1ab_0	conda-forge
icu	78.3	h33c6efd_0	conda-forge
idna	3.17	pyhcf101f3_0	conda-forge
igraph	1.0.1	hfe3e89f_0	conda-forge
importlib-metadata	9.0.0	pyhcf101f3_0	conda-forge
infernal	1.1.5	pl5321h7b50bb2_4	bioconda
intbitset	3.0.2	py311h459d7ec_1	conda-forge

```
iqtree                                  3.1.2             h8471819_0
bioconda
jinja2                                  3.1.6             pyhcf101f3_1
conda-forge
joblib                                  1.5.3             pyhd8ed1ab_0
conda-forge
kernel-headers_linux-64                 6.12.0            he073ed8_6
conda-forge
keyutils                                1.6.3             hb9d3cd8_0
conda-forge
kiwisolver                              1.5.0             py311h724c32c_0
conda-forge
krb5                                    1.22.2            ha1258a1_0
conda-forge
lcms2                                   2.19.1            h0c24ade_1
conda-forge
ld_impl_linux-64                        2.45.1            default_hbd61a6d_102
conda-forge
lerc                                    4.1.0             hdb68285_0
conda-forge
libasprintf                             0.25.1            h3f43e3d_1
conda-forge
libblas                                 3.11.0            8_h4a7cf45_openblas
conda-forge
libbrotlicommon                         1.2.0             hb03c661_1
conda-forge
libbrotlidec                            1.2.0             hb03c661_1
conda-forge
libbrotlienc                            1.2.0             hb03c661_1
conda-forge
libcblas                                3.11.0            8_h0358290_openblas
conda-forge
libcups                                 2.3.3             h7a8fb5f_6
conda-forge
libcurl                                 8.20.0            hcf29cc6_0
conda-forge
libdb                                   6.2.32            h9c3ff4c_0
conda-forge
libdeflate                              1.25              h17f619e_0
conda-forge
libedit                                 3.1.20250104      pl5321h7949ede_0
conda-forge
libev                                   4.33              hd590300_2
conda-forge
libexpat                                2.8.1             hecca717_0
conda-forge
libffi                                  3.5.2             h3435931_0
conda-forge
libfreetype                             2.14.3            ha770c72_0
conda-forge
libfreetype6                            2.14.3            h73754d4_0
conda-forge
libgcc                                  15.2.0            he0feb66_19
conda-forge
```

libgcc-devel_linux-64	15.2.0	hcc6f6b0_119	conda-forge
libgcc-ng	15.2.0	h69a702a_19	conda-forge
libgettextpo	0.25.1	h3f43e3d_1	conda-forge
libgfortran	15.2.0	h69a702a_19	conda-forge
libgfortran5	15.2.0	h68bc16d_19	conda-forge
libglib	2.88.1	h0d30a3d_2	conda-forge
libgomp	15.2.0	he0feb66_19	conda-forge
libiconv	1.18	h3b78370_2	conda-forge
libidn11	1.34	h1cef754_0	conda-forge
libidn2	2.3.8	hfac485b_1	conda-forge
libjpeg-turbo	3.1.4.1	hb03c661_0	conda-forge
liblapack	3.11.0	8_h47877c9_openblas	conda-forge
liblzma	5.8.3	hb03c661_0	conda-forge
liblzma-devel	5.8.3	hb03c661_0	conda-forge
libnghttp2	1.68.1	h877daf1_0	conda-forge
libnsl	2.0.1	hb9d3cd8_1	conda-forge
libopenblas	0.3.33	pthreads_h94d23a6_0	conda-forge
libpng	1.6.58	h421ea60_0	conda-forge
libsanitizer	15.2.0	h90f66d4_19	conda-forge
libsqlite	3.53.2	h0c1763c_0	conda-forge
libssh2	1.11.1	hcf80075_0	conda-forge
libstdcxx	15.2.0	h934c35e_19	conda-forge
libstdcxx-devel_linux-64	15.2.0	hd446a21_119	conda-forge
libstdcxx-ng	15.2.0	hdf11a46_19	conda-forge
libtasn1	4.21.0	hb03c661_0	conda-forge
libtiff	4.7.1	h9d88235_1	conda-forge
libunistring	0.9.10	h7f98852_0	conda-forge

```
libuuid                                 2.42.1           h5347b49_0
conda-forge
libuv                                   1.52.1           h280c20c_0
conda-forge
libwebp-base                            1.6.0            hd42ef1d_0
conda-forge
libxcb                                  1.17.0           h8a09558_0
conda-forge
libxcrypt                               4.4.36           hd590300_1
conda-forge
libxml2                                 2.15.3           h49c6c72_0
conda-forge
libxml2-16                              2.15.3           hca6bf5a_0
conda-forge
libxml2-devel                           2.15.3           h49c6c72_0
conda-forge
libzlib                                 1.3.2            h25fd6f3_2
conda-forge
llvmlite                                0.47.0           py311h41a00d4_1
conda-forge
mafft                                   7.526            h4bc722e_0
conda-forge
make                                    4.4.1            hb9d3cd8_2
conda-forge
markupsafe                              3.0.3            py311h3778330_1
conda-forge
mash                                    2.3              hb105d93_9
bioconda
mashtree                                1.4.6            pl5321h87e0c26_4
bioconda
matplotlib-base                         3.10.9           py311h0f3be63_0
conda-forge
minced                                  0.4.2            hdfd78af_1
bioconda
mmseqs2                                 18.8cc5c         hd6d6fdc_0
bioconda
mpfr                                    4.2.2            he0a73b1_0
conda-forge
mummer4                                 4.0.1            pl5321h9948957_0
bioconda
munkres                                 1.1.4            pyhd8ed1ab_1
conda-forge
muscle                                  5.3              h9948957_3
bioconda
narwhals                                2.22.0           pyhcf101f3_0
conda-forge
ncbi-vdb                                3.4.1            hd63eeec_0
bioconda
ncurses                                 6.6              hdb14827_0
conda-forge
nettle                                  3.10.1           h4a9d5aa_0
conda-forge
networkx                                3.6.1            pyhcf101f3_0
conda-forge
```

numba	0.65.1	py311h3c884d5_1	conda-forge
numpy	2.4.6	py311h2e04523_0	conda-forge
openjdk	25.0.2	ha668962_0	conda-forge
openjpeg	2.5.4	h55fea9a_0	conda-forge
openssl	3.6.2	h35e630c_0	conda-forge
p11-kit	0.26.2	h3435931_0	conda-forge
packaging	26.2	pyhc364b38_0	conda-forge
paml	4.10.10	h87e0c26_2	bioconda
panaroo	1.7.0	pyhdfd78af_0	bioconda
pandoc	3.10	ha770c72_0	conda-forge
pango	1.56.4	hda50119_1	conda-forge
parallel	20260422	ha770c72_0	conda-forge
pcre2	10.47	haa7fec5_0	conda-forge
peewee	4.0.6	py311h8e11ec1_0	conda-forge
perl	5.32.1	7_hd590300_perl5	conda-forge
perl-algorithm-diff	1.201	pl5321hd8ed1ab_0	conda-forge
perl-archive-tar	3.10	pl5321hdfd78af_0	bioconda
perl-b-cow	0.007	pl5321hb9d3cd8_1	conda-forge
perl-base	2.23	pl5321hd8ed1ab_0	conda-forge
perl-bio-asn1-entrezgene	1.73	pl5321hdfd78af_3	bioconda
perl-bio-coordinate	1.007001	pl5321hdfd78af_3	bioconda
perl-bio-featureio	1.6.905	pl5321hdfd78af_4	bioconda
perl-bio-samtools	1.43	pl5321h577a1d6_6	bioconda
perl-bio-searchio-hmmer	1.7.3	pl5321hdfd78af_0	bioconda
perl-bio-tools-phylo-paml	1.7.3	pl5321hdfd78af_3	bioconda
perl-bio-tools-run-alignment-clustalw	1.7.4	pl5321hdfd78af_3	bioconda
perl-bio-tools-run-alignment-tcoffee	1.7.4	pl5321hdfd78af_5	bioconda

perl-bioperl	1.7.8	hdfd78af_1	bioconda
perl-bioperl-core	1.7.8	pl5321hdfd78af_1	bioconda
perl-bioperl-run	1.007003	pl5321hdfd78af_0	bioconda
perl-business-isbn	3.007	pl5321hd8ed1ab_0	conda-forge
perl-business-isbn-data	20210112.006	pl5321hd8ed1ab_0	conda-forge
perl-carp	1.50	pl5321hd8ed1ab_0	conda-forge
perl-class-data-inheritable	0.09	pl5321ha770c72_0	conda-forge
perl-clone	0.46	pl5321hb9d3cd8_1	conda-forge
perl-common-sense	3.75	pl5321hd8ed1ab_0	conda-forge
perl-compress-raw-bzip2	2.214	pl5321hda65f42_0	conda-forge
perl-compress-raw-zlib	2.214	pl5321h4dac143_0	conda-forge
perl-constant	1.33	pl5321hd8ed1ab_0	conda-forge
perl-data-dump	1.25	pl5321h7b50bb2_2	bioconda
perl-data-dumper	2.183	pl5321hb9d3cd8_1	conda-forge
perl-db_file	1.858	pl5321hb9d3cd8_0	conda-forge
perl-devel-stacktrace	2.04	pl5321h296ab09_0	conda-forge
perl-digest-hmac	1.05	pl5321hdfd78af_0	bioconda
perl-digest-md5	2.59	pl5321hb9d3cd8_3	conda-forge
perl-encode	3.24	pl5321hb03c661_0	conda-forge
perl-encode-locale	1.05	pl5321hdfd78af_7	bioconda
perl-exception-class	1.45	pl5321ha770c72_0	conda-forge
perl-exporter	5.74	pl5321hd8ed1ab_0	conda-forge
perl-exporter-tiny	1.002002	pl5321hd8ed1ab_0	conda-forge
perl-extutils-makemaker	7.70	pl5321hd8ed1ab_0	conda-forge
perl-file-listing	6.16	pl5321hdfd78af_0	bioconda
perl-file-path	2.18	pl5321hd8ed1ab_0	conda-forge
perl-file-slurper	0.014	pl5321hdfd78af_0	bioconda

```
perl-file-sort                          1.01            pl5321hdfd78af_3
bioconda
perl-file-spec                          3.48_01         pl5321hdfd78af_2
bioconda
perl-file-temp                          0.2304          pl5321hd8ed1ab_0
conda-forge
perl-file-which                         1.24            pl5321hd8ed1ab_0
conda-forge
perl-getopt-long                        2.58            pl5321hdfd78af_0
bioconda
perl-html-parser                        3.81            pl5321h4ac6f70_1
bioconda
perl-html-tagset                        3.24            pl5321hdfd78af_0
bioconda
perl-http-cookiejar-lwp                 0.014           pl5321hdfd78af_0
bioconda
perl-http-cookies                       6.11            pl5321hdfd78af_0
bioconda
perl-http-daemon                        6.17            pl5321hdfd78af_0
bioconda
perl-http-date                          6.06            pl5321hdfd78af_0
bioconda
perl-http-message                       7.01            pl5321hdfd78af_0
bioconda
perl-http-negotiate                     6.01            pl5321hdfd78af_4
bioconda
perl-inc-latest                         0.500           pl5321ha770c72_0
conda-forge
perl-io-compress                        2.216           pl5321h503566f_0
bioconda
perl-io-html                            1.004           pl5321hdfd78af_0
bioconda
perl-io-socket-ssl                      2.075           pl5321hd8ed1ab_0
conda-forge
perl-io-string                          1.08            pl5321hdfd78af_4
bioconda
perl-io-tty                             1.31            pl5321hb03c661_0
conda-forge
perl-io-zlib                            1.15            pl5321hdfd78af_1
bioconda
perl-ipc-run                            20250809.0      pl5321hdfd78af_0
bioconda
perl-json                               4.11            pl5321hdfd78af_0
bioconda
perl-json-xs                            4.04            pl5321h9948957_0
bioconda
perl-libwww-perl                        6.82            pl5321hdfd78af_0
bioconda
perl-libxml-perl                        0.08            pl5321hdfd78af_3
bioconda
perl-list-moreutils                     0.430           pl5321hdfd78af_0
bioconda
perl-list-moreutils-xs                  0.430           pl5321h7b50bb2_5
bioconda
```

perl-lwp-mediatypes	6.04	pl5321hdfd78af_1	bioconda
perl-lwp-protocol-https	6.15	pl5321hdfd78af_0	bioconda
perl-mime-base64	3.16	pl5321hb9d3cd8_3	conda-forge
perl-module-build	0.4234	pl5321ha770c72_1	conda-forge
perl-module-load	0.34	pl5321hdfd78af_0	bioconda
perl-mozilla-ca	20250602	pl5321hdfd78af_0	bioconda
perl-net-http	6.24	pl5321hdfd78af_0	bioconda
perl-net-ssleay	1.96	pl5321h34fdc24_0	conda-forge
perl-ntlm	1.09	pl5321hdfd78af_5	bioconda
perl-parent	0.243	pl5321hd8ed1ab_0	conda-forge
perl-path-tiny	0.150	pl5321hd8ed1ab_0	conda-forge
perl-pathtools	3.75	pl5321hb9d3cd8_2	conda-forge
perl-scalar-list-utils	1.70	pl5321hb03c661_0	conda-forge
perl-socket	2.027	pl5321h5c03b87_6	bioconda
perl-storable	3.15	pl5321hb9d3cd8_2	conda-forge
perl-sub-uplevel	0.2800	pl5321hb9d3cd8_0	conda-forge
perl-test-deep	1.130	pl5321hd8ed1ab_0	conda-forge
perl-test-differences	0.72	pl5321ha770c72_0	conda-forge
perl-test-exception	0.43	pl5321hd8ed1ab_0	conda-forge
perl-test-fatal	0.016	pl5321hdfd78af_0	bioconda
perl-test-most	0.42	pl5321hdfd78af_0	bioconda
perl-test-needs	0.002009	pl5321hd8ed1ab_0	conda-forge
perl-test-requiresinternet	0.05	pl5321hdfd78af_1	bioconda
perl-test-warn	0.37	pl5321hd8ed1ab_0	conda-forge
perl-test-warnings	0.031	pl5321ha770c72_0	conda-forge
perl-text-diff	1.45	pl5321hd8ed1ab_0	conda-forge
perl-time-local	1.35	pl5321hdfd78af_0	bioconda

perl-timedate	2.35	pl5321hdfd78af_0
bioconda		
perl-tree-dag_node	1.35	pl5321hdfd78af_0
bioconda		
perl-try-tiny	0.32	pl5321ha770c72_1
conda-forge		
perl-types-serialiser	1.01	pl5321hdfd78af_0
bioconda		
perl-uri	5.34	pl5321ha770c72_0
conda-forge		
perl-url-encode	0.03	pl5321h9ee0642_1
bioconda		
perl-www-robotrules	6.03	pl5321hdfd78af_0
bioconda		
perl-xml-dom	1.46	pl5321hdfd78af_1
bioconda		
perl-xml-dom-xpath	0.14	pl5321hdfd78af_2
bioconda		
perl-xml-namespacesupport	1.12	pl5321hd8ed1ab_0
conda-forge		
perl-xml-parser	2.44_01	pl5321hf2e2c51_1004
conda-forge		
perl-xml-regexp	0.04	pl5321hdfd78af_3
bioconda		
perl-xml-sax	1.02	pl5321hd8ed1ab_0
conda-forge		
perl-xml-sax-base	1.09	pl5321hd8ed1ab_0
conda-forge		
perl-xml-sax-expat	0.51	pl5321hd8ed1ab_0
conda-forge		
perl-xml-simple	2.25	pl5321hdfd78af_2
bioconda		
perl-xml-xpathengine	0.14	pl5321hdfd78af_3
bioconda		
pillow	12.2.0	py311hf88fc01_0
conda-forge		
pip	26.1.2	pyh8b19718_0
conda-forge		
pixman	0.46.4	h54a6638_1
conda-forge		
plotly	6.8.0	pyhd8ed1ab_0
conda-forge		
poa	2.0	h7b50bb2_6
bioconda		
prank	251117	hd63eeec_0
bioconda		
prodigal	2.6.3	h577a1d6_11
bioconda		
prokka	1.15.6	pl5321hdfd78af_0
bioconda		
pthread-stubs	0.4	hb9d3cd8_1002
conda-forge		
pyfaidx	0.9.0.4	pyhdfd78af_0
bioconda		

pyparsing	3.3.2	pyhcf101f3_0	conda-forge
pyrodigal	3.7.1	py311h26ae33e_1	bioconda
pysocks	1.7.1	pyha55dd90_7	conda-forge
python	3.11.15	hd63d673_0_cpython	conda-forge
python-dateutil	2.9.0.post0	pyhe01879c_2	conda-forge
python-edlib	1.3.9.post1	py311hf552afe_3	bioconda
python-igraph	1.0.0	py311h1ddb823_0	conda-forge
python_abi	3.11	8_cp311	conda-forge
pyvcf3	1.0.4	py311haab0aaa_0	bioconda
qhull	2020.2	h434a139_5	conda-forge
quicktree	2.5	h7b50bb2_9	bioconda
r-ape	5.8_1	r45h3704496_2	conda-forge
r-aplot	0.2.9	r45hc72bb7e_1	conda-forge
r-base	4.5.3	h502d0c9_2	conda-forge
r-base64enc	0.1_6	r45h54b55ab_0	conda-forge
r-bslib	0.11.0	r45hc72bb7e_0	conda-forge
r-cachem	1.1.0	r45h54b55ab_2	conda-forge
r-cli	3.6.6	r45h3697838_0	conda-forge
r-colorspace	2.1_2	r45h54b55ab_0	conda-forge
r-commonmark	2.0.0	r45h54b55ab_1	conda-forge
r-cpp11	0.5.5	r45h785f33e_0	conda-forge
r-crayon	1.5.3	r45hc72bb7e_2	conda-forge
r-crul	1.6.0	r45hc72bb7e_1	conda-forge
r-curl	7.1.0	r45h10955f1_0	conda-forge
r-digest	0.6.39	r45h3697838_0	conda-forge
r-dplyr	1.2.1	r45h3697838_0	conda-forge
r-ellipsis	0.3.3	r45h54b55ab_0	conda-forge

```
r-evaluate                              1.0.5            r45hc72bb7e_1
conda-forge
r-fansi                                 1.0.7            r45h54b55ab_0
conda-forge
r-farver                                2.1.2            r45h3697838_2
conda-forge
r-fastmap                               1.2.0            r45h3697838_2
conda-forge
r-fontawesome                           0.5.3            r45hc72bb7e_1
conda-forge
r-fontbitstreamvera                     0.1.1            r45hc72bb7e_1007
conda-forge
r-fontliberation                        0.1.0            r45hc72bb7e_1007
conda-forge
r-fontquiver                            0.2.1            r45hc72bb7e_1007
conda-forge
r-fs                                    2.1.0            r45h3697838_0
conda-forge
r-gdtools                               0.5.1            r45ha0c56d8_0
conda-forge
r-generics                              0.1.4            r45hc72bb7e_1
conda-forge
r-gfonts                                0.2.0            r45hc72bb7e_3
conda-forge
r-ggfun                                 0.2.0            r45hc72bb7e_1
conda-forge
r-ggiraph                               0.9.6            r45h8ff94db_0
conda-forge
r-ggplot2                               4.0.3            r45h785f33e_0
conda-forge
r-ggplotify                             0.1.3            r45hc72bb7e_1
conda-forge
r-glue                                  1.8.1            r45h54b55ab_0
conda-forge
r-gridgraphics                          0.5_1            r45hc72bb7e_4
conda-forge
r-gtable                                0.3.6            r45hc72bb7e_1
conda-forge
r-highr                                 0.12             r45hc72bb7e_0
conda-forge
r-htmltools                             0.5.9            r45h3697838_0
conda-forge
r-htmlwidgets                           1.6.4            r45h785f33e_4
conda-forge
r-httpcode                              0.3.0            r45ha770c72_5
conda-forge
r-httpuv                                1.6.17           r45h6d565e7_0
conda-forge
r-isoband                               0.3.0            r45h3697838_0
conda-forge
r-jquerylib                             0.1.4            r45hc72bb7e_4
conda-forge
r-jsonlite                              2.0.0            r45h54b55ab_1
conda-forge
```

```
r-knitr                                   1.51             r45hc72bb7e_0
conda-forge
r-labeling                                0.4.3            r45hc72bb7e_2
conda-forge
r-later                                   1.4.8            r45h3697838_0
conda-forge
r-lattice                                 0.22_9           r45h54b55ab_0
conda-forge
r-lazyeval                                0.2.3            r45h54b55ab_0
conda-forge
r-lifecycle                               1.0.5            r45hc72bb7e_0
conda-forge
r-magrittr                                2.0.5            r45h54b55ab_0
conda-forge
r-mass                                    7.3_65           r45h54b55ab_0
conda-forge
r-memoise                                 2.0.1            r45hc72bb7e_4
conda-forge
r-mime                                    0.13             r45h54b55ab_1
conda-forge
r-munsell                                 0.5.1            r45hc72bb7e_2
conda-forge
r-nlme                                    3.1_169          r45heaba542_0
conda-forge
r-otel                                    0.2.0            r45hc72bb7e_1
conda-forge
r-patchwork                               1.3.2            r45hc72bb7e_1
conda-forge
r-pillar                                  1.11.1           r45hc72bb7e_0
conda-forge
r-pkgconfig                               2.0.3            r45hc72bb7e_5
conda-forge
r-promises                                1.5.0            r45hc72bb7e_1
conda-forge
r-purrr                                   1.2.2            r45h54b55ab_0
conda-forge
r-r6                                      2.6.1            r45hc72bb7e_1
conda-forge
r-rappdirs                                0.3.4            r45h54b55ab_0
conda-forge
r-rcolorbrewer                            1.1_3            r45h785f33e_4
conda-forge
r-rcpp                                    1.1.1_1.1        r45h3697838_0
conda-forge
r-rlang                                   1.2.0            r45h3697838_0
conda-forge
r-rmarkdown                               2.31             r45hc72bb7e_0
conda-forge
r-s7                                      0.2.2            r45h54b55ab_0
conda-forge
r-sass                                    0.4.10           r45h3697838_1
conda-forge
r-scales                                  1.4.0            r45hc72bb7e_1
conda-forge
```

```
r-shiny                                   1.13.0          r45h785f33e_0
conda-forge
r-sourcetools                             0.1.7_2         r45h3697838_0
conda-forge
r-stringi                                 1.8.7           r45h3d52c89_2
conda-forge
r-stringr                                 1.6.0           r45h785f33e_0
conda-forge
r-systemfonts                             1.3.2           r45h74f4acd_0
conda-forge
r-tibble                                  3.3.1           r45h54b55ab_0
conda-forge
r-tidyr                                   1.3.2           r45h3697838_0
conda-forge
r-tidyselect                              1.2.1           r45hc72bb7e_2
conda-forge
r-tidytree                                0.4.7           r45hc72bb7e_0
conda-forge
r-tinytex                                 0.59            r45hc72bb7e_0
conda-forge
r-triebeard                               0.4.1           r45h3697838_4
conda-forge
r-urltools                                1.7.3.1         r45h3697838_1
conda-forge
r-utf8                                    1.2.6           r45h54b55ab_1
conda-forge
r-vctrs                                   0.7.3           r45h3697838_0
conda-forge
r-viridislite                             0.4.3           r45hc72bb7e_0
conda-forge
r-withr                                   3.0.2           r45hc72bb7e_1
conda-forge
r-xfun                                    0.57            r45h3697838_0
conda-forge
r-xtable                                  1.8_8           r45hc72bb7e_0
conda-forge
r-yaml                                    2.3.12          r45h54b55ab_0
conda-forge
r-yulab.utils                             0.2.4           r45hc72bb7e_0
conda-forge
readline                                  8.3             h853b02a_0
conda-forge
requests                                  2.34.2          pyhcf101f3_0
conda-forge
ruby                                      4.0.5           h95e6935_0
conda-forge
samtools                                  0.1.19          h9dcdb79_15
bioconda
scikit-learn                              1.9.0           np2py311ha15b03d_0
conda-forge
scipy                                     1.17.1          py311hbe70eeb_1
conda-forge
sed                                       4.10            h19d0853_0
conda-forge
```

seqkit	2.13.0	he881be0_0	bioconda
setuptools	82.0.1	pyh332efcf_0	conda-forge
simplejson	4.1.1	py311h49ec1c0_0	conda-forge
six	1.17.0	pyhe01879c_1	conda-forge
sysroot_linux-64	2.39	hc4b9eeb_6	conda-forge
t-coffee	12.00.7fb08c2	h26a2512_0	bioconda
taxadb	0.12.1	pyh5e36f6f_0	bioconda
taxonkit	0.20.0	h9ee0642_1	bioconda
tbl2asn-forever	25.7.2f	h7b50bb2_6	bioconda
texttable	1.7.0	pyhd8ed1ab_1	conda-forge
threadpoolctl	3.6.0	pyhecae5ae_0	conda-forge
tk	8.6.13	noxft_h366c992_103	conda-forge
tktable	2.10	h5a7a40f_8	conda-forge
tqdm	4.67.3	pyh8f84b5b_0	conda-forge
transtermhp	2.09	h9948957_2	bioconda
tzdata	2025c	hc9c84f9_1	conda-forge
unicodedata2	17.0.1	py311h49ec1c0_0	conda-forge
unzip	6.0	hb03c661_4	conda-forge
urllib3	2.7.0	pyhd8ed1ab_0	conda-forge
viennarna	2.6.4	py311pl5321h6eedab3_2	bioconda
wget	1.25.0	h653f8fd_1	conda-forge
wheel	0.47.0	pyhd8ed1ab_0	conda-forge
xorg-libice	1.1.2	hb9d3cd8_0	conda-forge
xorg-libsm	1.2.6	he73a12e_0	conda-forge
xorg-libx11	1.8.13	he1eb515_0	conda-forge
xorg-libxau	1.0.12	hb03c661_1	conda-forge
xorg-libxdmcp	1.1.5	hb03c661_1	conda-forge

xorg-libxext	1.3.7	hb03c661_0	conda-forge
xorg-libxfixes	6.0.2	hb03c661_0	conda-forge
xorg-libxi	1.8.3	hb03c661_0	conda-forge
xorg-libxrandr	1.5.5	hb03c661_0	conda-forge
xorg-libxrender	0.9.12	hb9d3cd8_0	conda-forge
xorg-libxt	1.3.1	hb9d3cd8_0	conda-forge
xorg-libxtst	1.2.5	hb9d3cd8_3	conda-forge
xorg-xextproto	7.3.0	hb9d3cd8_1004	conda-forge
xz	5.8.3	ha02ee65_0	conda-forge
xz-gpl-tools	5.8.3	ha02ee65_0	conda-forge
xz-tools	5.8.3	hb03c661_0	conda-forge
yaggo	1.5.10	hdfd78af_1	bioconda
yaml	0.2.5	h280c20c_3	conda-forge
ziggypep	0.3.1	hdfd78af_0	bioconda
zipp	4.1.0	pyhcf101f3_0	conda-forge
zlib	1.3.2	h25fd6f3_2	conda-forge
zlib-ng	2.3.3	hceb46e0_1	conda-forge
zstd	1.5.7	hb78ec9c_6	conda-forge
